# Identification of Genes Controlled by the Essential YycFG Two-Component System Reveals a Role for Biofilm Modulation in *Staphylococcus epidermidis*

**DOI:** 10.3389/fmicb.2017.00724

**Published:** 2017-04-26

**Authors:** Tao Xu, Yang Wu, Zhiwei Lin, Ralph Bertram, Friedrich Götz, Ying Zhang, Di Qu

**Affiliations:** ^1^Key Laboratory of Medical Molecular Virology of Ministries of Education and Health, Department of Medical Microbiology and Parasitology, Institute of Medical Microbiology and Institutes of Biomedical Sciences, Fudan UniversityShanghai, China; ^2^Key Laboratory of Medical Molecular Virology, Huashan Hospital, Shanghai Medical College of Fudan UniversityShanghai, China; ^3^Klinikum Nürnberg Medical School GmbH, Research Department, Paracelsus Medical UniversityNuremberg, Germany; ^4^Department of Microbial Genetics, Faculty of Science, Interfaculty Institute of Microbiology and Infection Medicine Tübingen, University of TübingenTübingen, Germany; ^5^Department of Molecular Microbiology and Immunology, Bloomberg School of Public Health, Johns Hopkins UniversityBaltimore, MD, USA

**Keywords:** *Staphylococcus epidermidis*, biofilm, YycFG, two-component signal transduction system, antisense RNA

## Abstract

Biofilms play a crucial role in the pathogenicity of *Staphylococcus epidermidis*, while little is known about whether the essential YycFG two-component signal transduction system (TCS) is involved in biofilm formation. We used antisense RNA (asRNA) to silence the *yycFG* TCS in order to study its regulatory functions in *S. epidermidis*. Strain 1457 expressing asRNA_*yycF*_ exhibited a significant delay (~4–5 h) in entry to log phase, which was partially complemented by overexpressing *ssaA*. The expression of asRNA_*yycF*_ and asRNA_*yycG*_ resulted in a 68 and 50% decrease in biofilm formation at 6 h, respectively, while they had no significant inhibitory effect on 12 h biofilm formation. The expression of asRNA_*yycF*_ led to a ~5-fold increase in polysaccharide intercellular adhesion (PIA) production, but it did not affect the expression of accumulation-associated protein (Aap) or the release of extracellular DNA. Consistently, quantitative real-time PCR showed that silencing *yycF* resulted in an increased transcription of biofilm-related genes, including *icaA, arlR, sarA, sarX*, and *sbp*. An *in silico* search of the YycF regulon for the conserved YycF recognition pattern and a modified motif in *S. epidermidis*, along with additional gel shift and DNase I footprinting assays, showed that *arlR, sarA, sarX*, and *icaA* are directly regulated by YycF. Our data suggests that YycFG modulates *S. epidermidis* biofilm formation in an ica-dependent manner.

## Introduction

The coagulase-negative *Staphylococcus epidermidis*, an opportunistic pathogen, has become the most common source of infections associated with indwelling medical devices (Simon et al., 2005; Gordon et al., 2006). The pathogenicity of *S. epidermidis* is mainly attributed to biofilm formation, which involves multiple matrix components and regulators (Fey and Olson, 2010; Flemming et al., 2016).

Biofilm formation is a phased process that includes initial adhesion, proliferation, and detachment (Otto, 2009; Mack et al., 2013). The matrix of the three-dimensional structured staphylococcal biofilm is mainly composed of extracellular polymeric substances (EPS), which includes polysaccharide intercellular adhesion (PIA) (O'Gara, 2007) and extracellular DNA (eDNA) (Qin et al., 2007). PIA, the major component of staphylococcal biofilm, is synthesized by proteins encoded by *icaADBC*, which are negatively regulated by the transcriptional repressor IcaR (Jefferson et al., 2003). In addition, eDNA released from cells plays an important role in biofilm formation. We previously reported that AtlE is one of the major murein hydrolases that mediate eDNA release (Qin et al., 2007). Extracellular proteins including accumulation-associated protein (Aap), biofilm-associated protein (Bap/Bhp), and extracellular matrix-binding protein (Embp) also participate in biofilm formation, especially in *ica*-independent pathways (Vandecasteele et al., 2003; Lasa and Penades, 2006; Christner et al., 2012). In *S. epidermidis*, Aap is a major component of protein-dependent biofilm formation (Conrady et al., 2008), and a recent study revealed that a Small basic protein (Sbp) serves as an essential scaffold between the B domains of two Aap molecules during cell aggregation (Decker et al., 2015).

Regulation of staphylococcal biofilm formation involves multiple transcriptional regulators that form a complex network. In addition to transcriptional regulators such as IcaR, SarA (Tormo et al., 2005), SarX (Rowe et al., 2011), and SarZ (Wang et al., 2008) that function as single regulators (Knobloch et al., 2001), TCSs play important roles in *S. epidermidis* biofilm formation. Our previous work showed that depletion of the ArlRS (Wu et al., 2012) or SrrAB (Wu et al., 2015) TCS impaired biofilm formation in *S. epidermidis*. (Howell et al., 2003; Botella et al., 2011; Fukushima et al., 2011; Delaune et al., 2012; Dhiman et al., 2014). It was noticed that the YycFG (also known as WalKR and VicKR) TCS is involved in biofilm formation in *S. aureus* (Dubrac et al., 2007), but its regulatory role in *S. epidermidis* biofilm formation has not previously been confirmed.

The highly conserved YycFG TCS is of noticeable importance because of its essentiality and regulatory roles in metabolism, cell division, cell wall synthesis, autolysis, and virulence (Howell et al., 2003; Botella et al., 2011; Fukushima et al., 2011; Delaune et al., 2012; Dhiman et al., 2014). YycFG TCSs among Gram-positive bacterial species share similar sequences and operon structures that contain 3–6 genes (Dubrac et al., 2008). In *S. epidermidis*, the *yyc* operon includes four genes, *yycF, yycG, yycH*, and *yycI*. YycG, the histidine kinase (HK), is anchored to the cell membrane, where it senses and transduces environmental signals. YycH and YycI are involved in the activation of YycG in *B. subtilis* and *S. aureus* (Santelli et al., 2007; Szurmant et al., 2008; Cameron et al., 2016). YycF is a typical OmpR family protein, serving as a response regulator (RR). When YycF is activated via phosphorylation by YycG, it binds the promoters of target genes based on a conserved pattern that is based on the recognition of a motif sequence [5′-TGT(A/T)A(A/T/C)-N5-TGT(A/T)A(A/T/C)-3′] by the helix-turn-helix domain of YycF. The pattern was first identified in *B. subtilis* (Howell et al., 2003) and later in *S. aureus* (Dubrac and Msadek, 2004) and *Streptococcus mutans* (Senadheera et al., 2005). YycFG TCS regulates biological processes by controlling the expression of various regulons among species (Dubrac and Msadek, 2008; Winkler and Hoch, 2008).

Since YycFG TCS is an essential element, creating a gene deletion mutant using homologous recombination was not possible. Therefore, antisense RNA (asRNA), which is able to silence target gene expression by stimulating sequence-specific mRNA degradation (Wagner and Simons, 1994; Bai et al., 2012), was used to investigate the functions of YycFG. We investigated the impacts of asRNA_*yycF*_ or asRNA_*yycG*_ on bacterial growth and biofilm formation in *S. epidermidis*. In addition, by carrying out an *in silico* search for the conserved and modified motif patterns in the YycF regulon of *S. epidermidis*, we identified YycFG target genes involved in energy production, translation, and cell wall metabolism, as well as biofilm formation. The role of YycF in the regulation of biofilm-related genes was confirmed. This study extends our understanding of the regulatory mechanisms involved in *S. epidermidis* biofilm formation, in which YycFG TCS plays an important role.

## Materials and methods

### Bacterial strains and culturing mediums

In this study, the *S. epidermidis* strain 1457 (SE1457) (Mack et al., 1992) was used as a wild type strain for gene silencing. *Escherichia coli* DC10B (Monk et al., 2012) was used to isolate shuttle plasmids for directly transforming *S. epidermidis* via electroporation (Lofblom et al., 2007). Lysogeny broth (LB) medium (1% tryptone, 0.5% yeast extract, and 0.5% NaCl) was used for the cultivation of *E. coli*. Basic medium (BM) (1% tryptone, 0.5% yeast extract, 0.5% glucose, 0.1% K_2_HPO_4_, and 0.5% NaCl) and tryptic soy broth (TSB) (Oxoid, UK) were used for *S. epidermidis* cultivation and biofilm formation. B2 media (1% tryptone, 2.5% yeast extract, 0.5% glucose, 0.1% K_2_HPO_4_, and 2.5% NaCl) was use for bacteria recovery after electroporation. Antibiotics were added at the following concentrations: chloramphenicol at 10 μg/ml and ampicillin at 100 μg/ml. Anhydrotetracycline (ATc, Sigma, USA) was used at a concentration of 250 ng/ml for induction of asRNA.

### Construction of asRNA plasmids

**Figure 1:**
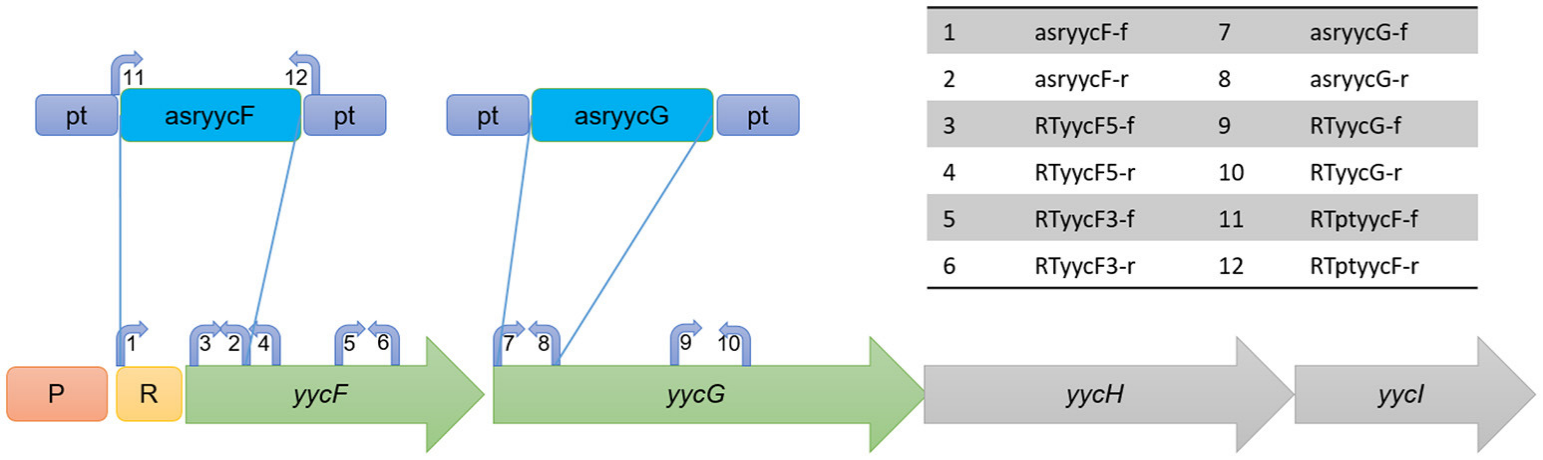
**Representation of structure of ***yyc*** operon and design of asRNA as well as primers**. The locations of the primers are indicated at the approximate locations on each gene. P, promoter of yyc operon; R, ribosome binding site.

To construct an asRNA expression vector, the paired termini 7 (PT7) segment (that can form a hairpin structure) was amplified using PCR with the primers prdtmn-f and prdtmn-r from plasmid pHN678 (Nakashima et al., 2006), digested with KpnI and SacI (Thermo Scientific, USA), and inserted into the ATc-inducible shuttle plasmid pALC2073 (Bateman et al., 2001). The resulting vector was named pMX6 (Figure 1A).

An expression plasmid of *yycF* asRNA (named pMXyycF) was constructed by first amplifying the predicted Shine-Dalgarno (SD) sequence plus ~100 nt downstream of the start codon of *yycF* and then inserting the fragment in the reverse direction between EagI and BglII sites downstream of the ATc-inducible promoter in pMX6. As for generating expression plasmids of *yycG* and *ssaA* asRNA, a ~120 nt sequence downstream from the start codon of each gene was amplified. All the asRNA expression plasmids were checked using DNA sequencing.

An overexpression plasmid of *ssaA* (named pMXyycF-ssaA) was constructed. The strong *sarA* P1 promoter of *S. aureus* strain 2395 was fused with the coding sequence of *S. epidermidis ssaA1* using PCR, and inserted at the SacI site of pMXyycF.

The primers used in this study are listed in Table 1.

**Table 1:** **Primers used in this study**.

**Primer**	**Sequence (5′ → 3′)**
**FOR CONSTRUCTION OF asRNA PLASMID**	
prdtmn-f	GGGGTACCTGGAATTGTGAGCGGATAAC
prdtmn-r	CGAGCTCCATGGGGTCAGGTGGGAC
asryycF-f	GAAGATCTGAACGGAAGAACTACACTTT
asryycF-r	AGCGGCCGCGCAATATACGTCGTAACC
asryycG-f	GAAGATCTATGAAGTGGCTTAAACAACTAC
asryycG-r	AGCGGCCGATTCCTTTTCTAAACTATTCG
asrssaA-f	GAAGATCTATGAAAAAAATCGCTACAGCTAC
asrssaA-r	AGCGGCCGGTGTAAGTGTAGCTATATGAATAAGGG
**FOR OVEREXPRESSION OF *ssaA1* (*serp1880*)**	
saraP1-f	CGGAGCTCCCTGATATTTTTGACTAAACCAAATGCT
saraP1-r	GATTTTTTTCATTGTTAAATATCCTCCTAAAAAGATGCATCTTGCTCGATAC
ssaA1-f	GTATCGAGCAAGATGCATCTTTTTAGGAGGATATTTAACAATGAAAAAAATC
ssaA1-r	CGGAGCTCCAGGTATTTGACTATGTTATACAAGTTTTATATG
**FOR PROTEIN EXPRESSION**	
REyycF-f	CGGATCCATGGCTAGAAAAGTTGTTGT
REyycF-r	CCCTCGAGCTAATCATGTTGTTGGAGGA
**FOR DETECTION OF eDNA**	
*gyrA-f*	CCTTATGAAACTCGGAGATGG
*gyrA-r*	TCAGTAGTAGTAGATTGTTGCG
*leuA-f*	GTGAACGGTATTGGTGAAAGAG
*leuA-r*	GTGGTCCTTCCTTACATATAAAGC
*lysA-f*	TGACAATGGGAGGTACAAGC
*lysA-r*	TGGTCTTCATCGTAAACAATCG
*serp0306-f*	ATGCCACATCCACGAAAGA
*serp0306-r*	TGTAACTGACAATGCCCAATC
**FOR DETECTION OF GENE EXPRESSION BY REAL-TIME PCR**	
RTgyrB-f	GCTAATGCCTCGTCAATAC
RTgyrB-r	TATGGTGCTGGACAGATAC
RTyycF5-f	GTTGTTGTAGTTGACGATGA
RTyycF5-r	ACGACATACTTCCATACCAT
RTyycF3-f	CCTGGTAGAGATGGTATGG
RTyycF3-r	AATAATGACGGCGTAAGTTC
RTptyycF-f	AATTTCAGGAGGAATTAACCAT
RTptyycF-r	TCATCGTCAACTACAACAAC
RTyycG-f	GCTTGGTGTCCTTAACTTAG
RTyycG-r	CGACTTGTTGTTGTTCTGT
RTyycH-f	ATTAGCCAACCATCCTGAT
RTyycH-r	TTGCCTTGTCGTCCATAT
RTyycI-f	ACAGCGATGGATGATATACA
RTyycI-r	CAGTTAGGTTGGAGTAATTGAA
RTrspA-f	GGCGATGTTATTGATGGTAA
RTrspA-r	CTACTGACACAACTTCTTCTG
RTssaA-f	GGAGTCCAGATCGTGTAA
RTssaA-r	TTGTGATTGCGTGTTGTT
RTaap-f	CGAGGAATTACAATCATCACA
RTaap-r	CGTAGTTGGCGGTATATCTA
RTicaR-f	GGAGCACTAGATAATTGAACAT
RTicaR-r	CATTGACGGACTTTACCAG
RTicaA-f	TCAAGCGAAGTCAATCTCT
RTicaA-r	AACAGCAATATCCTCAGTAATC
RTarlR-f	CTGTTGATATAGAGAATGATGGAA
RTarlR-r	TGATGATAATTGGAGTAGTTGTT
RTsarA-f	GTAATGAACACGATGAAAGAACT
RTsarA-r	GCTTCTGTGATACGGTTGT
RTsarX-f	CTGGCTACAGGAGAGTTAG
RTsarX-r	CAACATCTTCAAATAAAGCATCA
RTsbp-f	AAGATAGGCGAATCAATGAAG
RTsbp-r	GCGTGTAACATTTCCTCTT
**FOR PROMOTER AMPLIFICATION FOR EMSA**	
ProqoxB-f	TTACAAACCCCGTCATATCT
ProqoxB-r	TGCCAAATAATAGAAGCAAAG
PropitR-f	TGATTTTATCGCTCATCATTTT
PropitR-r	AAGTGGTTGAGGAACTGATT
PromurE-f	CACGATTTTAGTATTGTCTTC
PromurE-r	CACGTTCTATTAGATAGTGAT
PropstS-f	CATCTATTCATTCAATCAAGT
PropstS-r	ATATGTATTTCTTACAGTTCTC
ProsceD-f	ATGATAGGAATCATTACGGTT
ProsceD-r	CGTGCAGGTTACACTGAAA
ProssaA1-f	CACATTGCTATGTTAATTATTAT
ProssaA1-r	CGACAAGCCATACTCTAAC
ProrpsA-f	AAACTAAATTGACCATCACTT
ProrpsA-r	TATGCCTCCTTATACACTAC
ProarlR-f	ATAATGCTAGAGGGACTTTTT
ProarlR-r	CACCTCACGCTACATCTTA
Proica-f	TTCTAAAATCTCCCCCTTATTCAATTTTCT
Proica-r	TTTTTCACCTACCTTTCGTTAGTTAGGTTGT
Proaap-f	ATTATTCAAATGCTTGTAGT
Proaap-r	ATTATACCTCCCATGTATTT
prosarA-f	GAATATAGCAAATGCTACAT
ProsarA-R	TAATGAAACCTCCCTATTTATAT
ProsarX-f	GGTCAATTCTCACCAAGAG
ProsarX-r	CTTTCCCTCAGTCTTTTATG

### Detection of bacterial growth and biofilm formation

Bacterial strains were cultured at 37°C and growth curves were determined by measuring the optical density (OD) values at 600 nm, at 1 h intervals.

The biofilm formation of the bacteria was detected using a semi-quantitative microtiter plate assay with 96-well polystyrene plates (Corning, USA). Overnight cultures were diluted 1:200 in 200 μl TSB and cultured at 37°C for 6 or 12 h, with or without the addition of 250 ng/ml ATc. The planktonic culture was removed for detection of cell density at OD_600_. The biofilms on the bottom of wells were washed with phosphate-buffered saline (PBS), and fixed with 99% methanol. The fixed biofilms were stained with 2% (wt/vol) crystal violet, resolved with acetic acid (30%), and detected at 570 nm with a spectrophotometer (Beckman Coulter DTX880, Beckman Instruments, USA).

### Detection of bacterial primary attachment

The primary attachment of bacterial strains was detected according to a protocol developed by Heilmann et al. (1996). Briefly, an *S. epidermidis* strain with the plasmid pMX6 or pMXyycF was cultured with or without the induction of 250 ng/ml ATc at 37°C until the OD_600_ reached 0.6. After centrifugation, bacterial cells were resuspended in BM and the volume was adjusted until OD_600_ reached 0.1. The samples were inoculated into a 6-well polystyrene petri dish (BD, USA) and incubated at 30°C for 30 min. After being washed with PBS five times, the attached bacterial cells were observed using a light microscope (Nikon, Japan) and photographed.

### Detection of extracellular polymeric substances (EPS)

For detecting PIA production, *S. epidermidis* strains were cultivated to the exponential phase until the OD_600_ reached 0.6. The bacteria were centrifuged for 5 min at 4°C and resuspended in 0.5 M Ethylenediaminetetraacetic acid (EDTA, pH 8.0). After boiling for 5 min, the samples were centrifuged (13,000 g), and the supernatant was treated with 20 mg/ml proteinase K (Merck, Germany) for 3 h at 37°C. Proteinase K was then inactivated by raising the temperature to 100°C for 5 min. Five-fold serial dilutions of the cell extracts were transferred to a nitrocellulose membrane (Merck, Darmstadt, Germany) with a 96-well dot blot device (Biometra GmbH, Germany). The air-dried nitrocellulose membrane was blocked with 5% (wt/vol) skim milk, incubated with 3.2 mg/ml horseradish peroxidase (HRP)-labeled wheat germ agglutinin (WGA-HRP conjugate, Lectinotest Laboratory, Ukraine) for 1 h (Al Laham et al., 2007), and then 4-chloride-1-naphthol (Sigma, USA) was added as the substrate for the chromogenic detection of HRP activity.

For eDNA detection, the bacteria were cultured until the OD_600_ reached 0.6. After centrifugation, the supernatant was filtered using 0.22-μm syringe filters to remove the bacterial cell debris. The eDNA was extracted using phenol-chloroform-isoamyl that was diluted 1:10 in Tris-EDTA buffer, and it was quantified using quantitative polymerase chain reaction (qPCR) with SYBR Premix Ex-Taq (Takara, Japan) and primers for *gyrA, leuA*, and *lysA* (listed in Table 1).

### Detection of Aap by western blotting

The presence of Aap was assessed by western blotting with a monoclonal antibody, MAB_18B6_, that we developed to target *S. epidermidis* Aap (Hu et al., 2011). The bacteria were cultivated until the OD_600_ reached 0.6, collected by centrifugation, and treated with 0.1 mg/ml lysostaphin (Sigma, USA). The samples were centrifuged (20,000 g) at 4°C for 30 min. The supernatants were separated using 7% sodium dodecyl sulfate (SDS)-polyacrylamide gel electrophoresis (PAGE) and electro-transferred onto a 0.45-μm polyvinylidene fluoride (PVDF) membrane (Millipore, USA). The membrane was treated with 10 ng/ml MAB_18B6_ and HRP-conjugated goat anti-mouse IgG (Santa Cruz, USA) as a secondary antibody, and then visualized using an enhanced chemiluminescence western blotting system (Thermo Scientific).

### Detection of bacterial autolysis

Triton X-100 was used to induce the autolysis of the *S. epidermidis* strains, which was detected by following a protocol developed by Dubrac et al. (2007). In brief, the bacteria were cultured until the OD_600_ reached 0.6, and centrifuged (13,000 g) for 5 min at 4°C. The pellets were washed with distilled water, resuspended in 50 mM Tris-HCl (pH 7.2) with 0.05% (vol/vol) Triton X-100, and incubated at 37°C with shaking for 4 h. The bacterial cell autolysis was determined by measuring the OD_600_ absorbance every 30 min.

### Observation of bacteria morphology using transmission electron microscopy

The *S. epidermidis* strains were cultured in TSB medium until the OD_600_ reached 0.6, centrifuged and resuspended in 2.5% glutaraldehyde in Dulbecco's PBS. After fixation in osmium tetroxide, the samples were dehydrated with increasing alcohol concentrations and transferred onto an electron microscope grid covered with a carbon-coated Formvar film. The bacteria were stained with 1% (w/v) uranyl acetate-lead acetate and examined with an S-520 electron microscope (Hitachi, Japan).

### Purification of recombinant YycF

For the gel shift and DNase I footprinting assays, an YycF recombinant expression plasmid (named pETMG-yycF) was constructed. The *yycF* gene was amplified by PCR with the primers REyycF-f and REyycF-r (Table 1), and inserted into a pETMG plasmid at BamHI and XhoI sites (Hu et al., 2011). After transformation into BL21 (DE3), the bacteria were cultured in LB medium at 37°C for 4 h and incubated for another 4 h at 30°C with 1 mM isopropyl-1-thio-β-D-galactopyranoside. The cells were disrupted using sonication in lysis buffer (50 mM Tris-Cl and 300 mM NaCl, pH 8.0), and they were then centrifuged at 15,000 g for 30 min. The recombinant polyhistidine-tagged GB1-YycF protein in the supernatants was purified using affinity chromatography with an Ni-nitrilotriacetic acid column (Qiagen, Germany) and further purified using Superdex 75 gel filtration columns (GE Healthcare, USA).

### Gel shift assay

The gel shift assay was carried out using a protocol developed by Hellman and Fried (2007). The upstream regions of genes were amplified by PCR with the primers listed in Table 1, while a fragment located in the *yycF* coding region was used as the negative control. Different concentrations of r-YycF were mixed with 20 nM DNA fragments in a binding buffer (10 mM Tris, 25 mM KCl, 1 mM EDTA, 2.5 mM MgCl_2_, and 5% vol/vol glycerol, pH 8.0). After incubation at 37°C for 30 min, the samples were loaded onto 10% polyacrylamide native gels (with a 29:1 acrylamide: bisacrylamide ratio) for electrophoresis in a Tris-acetate-EDTA buffer at 10 V/cm for about 1.5 h. The gels were stained with Gel-Red (Biotium, USA) and detected using a Gel Doc EZ system (Bio-Rad, USA).

### DNase I footprinting assay

The DNase I footprinting assay was performed by following a protocol developed by Wang et al. (2012). Briefly, the promoters of the *arlR, ica*, and *pitR* genes were cloned into a pUC18B-T vector (Shanghai Biotechnology Corporation, China), and the plasmids were used as the template for preparation of fluorescein amidite (FAM)-labeled probes with the primers M13F and M13R (both FAM-labeled). The FAM-labeled probes were purified using Wizard SV Gel and a PCR Clean-Up System (Promega, Southampton, UK), and quantified using NanoDrop 2000C (Thermo Scientific). For the DNase I footprinting assay, 200 ng probes were incubated with different amounts of r-YycF in 40 μl of binding buffer at 30°C for 30 min. Subsequently, 10 μl DNase I (0.01 unit) (Promega, UK) and 100 nmol CaCl_2_ were added, incubated for 1 min at 25°C, and stopped using 140 μl DNase I stopping solution (200 mM unbuffered sodium acetate, 30 mM EDTA, and 0.15% SDS). The DNA samples extracted with phenol/chloroform and precipitated with ethanol, and the pellets were dissolved in 35 μl MilliQ water. The samples were loaded onto a device to carry out capillary electrophoresis, and data were collected using the GeneScan-500 LIZ dye Size Standard (Applied Biosystems, USA).

### RNA extraction and quantitative real-time (qRT)-PCR

For RNA extraction, the *S. epidermidis* strains were cultured at 37°C with shaking. For the detection of asRNA expression and the gene silencing efficiency, the bacteria were cultured for 6 or 12 h. For detecting the expression levels of biofilm-related genes, the bacteria were cultivated until the OD_600_ reached 0.6. The cell pellets were washed with ice-cold normal saline and then homogenized using 0.1-mm Zirconia-silica beads in a Mini-BeadBeater (Biospec, Bartlesville, USA) at a speed of 3,600 rpm for 40 s following cooling on ice for 1 min. This homogenization and cooling cycle was repeated five times, then the samples were centrifuged at 15,000 rpm and the bacterial RNA in the supernatant was purified using an RNeasy Mini kit (Qiagen) and quantified using an ND-1000 spectrophotometer (Nanodrop Technologies, Wilmington, USA). RNA samples that had a 260/280 ratio between 2.0 and 2.2 were reverse transcribed using an iScript cDNA synthesis kit (Bio-Rad) following the manufacturer's protocol. The mRNA levels were quantified by using qRT-PCR with SYBR green PCR reagents (Takara, Japan) and the primers listed in Table 1, with the housekeeping gene *gyrB* being used as an endogenous control. The amplification efficiency of all primer pairs were determined according to the standard curve with four magnitude of templates. The specificity of primer pairs was determined with melting curve. All the qRT-PCR experiments were carried out in triplicate and the relative gene expression data were analyzed using the 2^−ΔΔCT^ method (Livak and Schmittgen, 2001).

### Sequence analysis

A comparison of the protein sequence of YycF among various bacterial species was carried out using Clustal X 2.0 (http://www.clustal.org). The YycF regulon in the *S. epidermidis* RP62A genome was predicted using a bioinformatics analysis with a custom-made script on Perl and an online relational database (http://genolist.pasteur.fr).

Motif-based sequence analysis was performed online using Motif Discovery from the MEME (Multiple Em for Motif Elicitation) suite (http://meme-suite.org/).

## Results

### Silencing of YycFG TCS by asRNA

Since YycFG TCS is essential, we used asRNA technology to individually silence the expression of *yycF* and *yycG*. First, we constructed an ATc-inducible asRNA-expressing plasmid, pMX6, which contained a paired termini sequence for the formation of a hairpin structure that mediated the asRNA transcriptional termination (Supplementary Figure 1). For silencing *yycF* and *yycG*, the asRNA plasmids pMXyycF and pMXyycG were constructed. The asRNA_*yycF*_ was designed to target a sequence from the Shine-Dalgarno site to the 103rd nt of the *yycF* coding sequence, while the segment of asRNA_*yycG*_ was designed to target a sequence from the start codon to the 129th nt of *yycG* to avoid interference with *yycF* expression (Figure 1).

**Figure 2:**
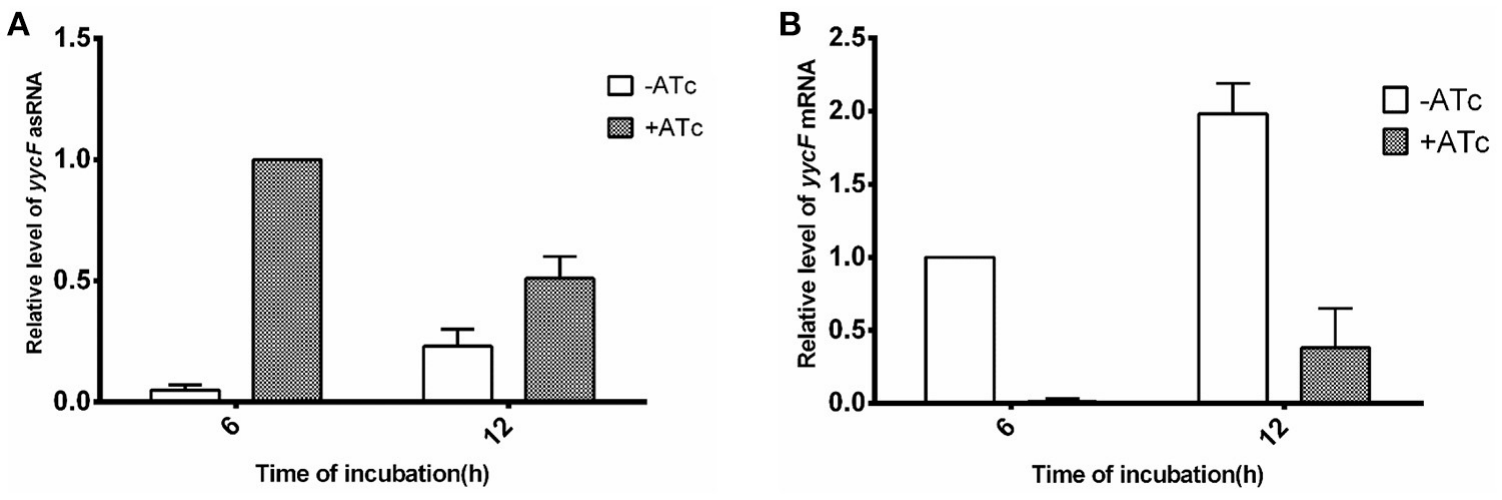
**Detection of asRNA_***yycF***_ and its effects on ***yycF*** mRNA**. SE1457 and its transformants with different plasmids were grown in BM for 6 or 12 h, with or without addition of ATc to 250 ng/ml. Total RNA was extracted and expression levels of asRNA **(A)** and mRNAs **(B)** were examined by qRT-PCR.

After transformation of the pMXyycF plasmid into SE1457, the expression levels of asRNA_*yycF*_ and *yycF* mRNA were quantified using qRT-PCR. In the bacteria that were incubated with 250 ng/ml ATc for 6 h, the transcription of asRNA_*yycF*_ was ~20-fold higher than that without ATc induction, but it decreased by ~50% at 12 h (Figure 2A). Correspondingly, the level of *yycF* mRNA when ATc was present was reduced by over 99% at 6 h compared to when ATc was not present, and it was ~75% lower at 12 h (Figure 2B). The similar time dependent trend was observed by detection of asRNA_*yycG*_ and *yycG* mRNA during induction of asRNA_*yycG*_ (Supplementary Figure 2).

### Effect of asRNA*_*yycF*_* or asRNA*_*yycG*_* on bacterial growth and cell morphology

**Figure 3:**
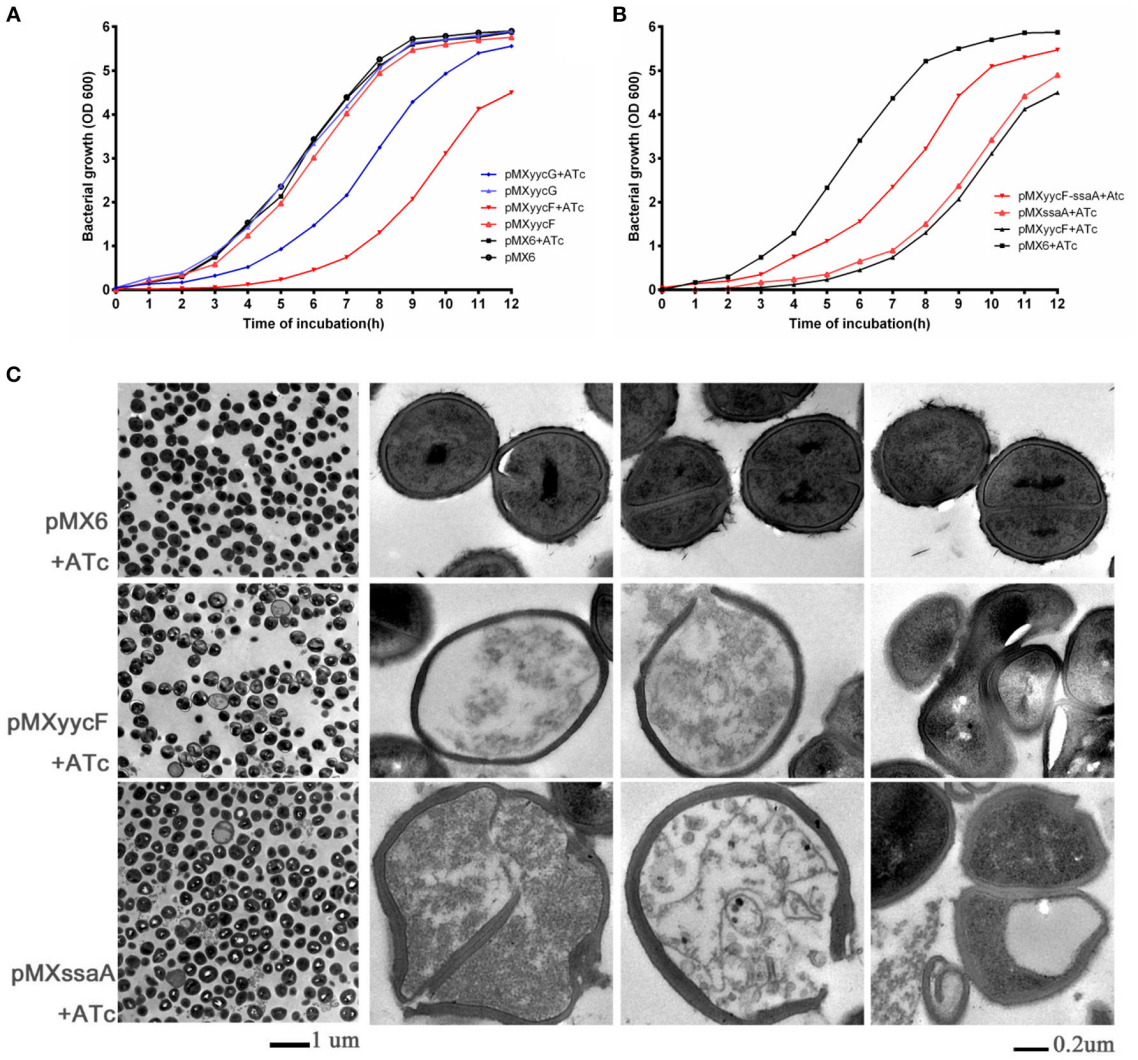
**Effects of asRNA on growth and morphology. (A)** Expression of *yycF* or *yycG* asRNA on bacterial growth. *S. epidermidis* 1457 with plasmids were grown in BM medium at 37°C, and growth was monitored every hour by measuring the turbidity of the cultures at OD_600_. **(B)** Effects of overexpression of *ssaA* on growth inhibition by asRNA_*yycF*_. The initial inoculation of each strain was 1:1,000 to optimize the effect of the asRNA. Similar results were obtained in three independent experiments. ATc, anhydrotetracycline (added to a final concentration of 250 ng/ml). **(C)** Transmission electron microscopy of effects from silencing of *yycF* and YycF target genes. SE 1457 strains were incubated in BM containing 10 μg/ml CM and 250 ng/ml ATc until an OD 600 of 0.6–0.8 was reached. From cells in pMX6, pMXyycF, and pMXssaA (first column on the left), cells with abnormal appearances from pMXyycF and pMXssaA were shown in the right 3 columns, while none was found in pMX6, which is the plasmid control. The white patches inside of some normal cells were probably due to insufficient penetration of EP612 resin into cell walls of gram positive bacteria.

The individual effects of asRNA_*yycF*_ and asRNA_*yycG*_ on bacterial growth were investigated. Under induction with 250 ng/ml ATc, the entrance into the log phase of SE1457 expressing asRNA_*yycF*_ was significantly delayed (~4–5 h) compared with non-ATc induction. However, the effect of asRNA_*yycG*_ on bacterial growth was weaker than the effect of asRNA_*yycF*_ (Figure 3A). Meanwhile, the addition of ATc did not affect the growth of the control strain, SE1457 containing the pMX6 vector.

In *S. aureus*, the indispensability of the YycFG TCS can be circumvented by overexpressing two autolysin genes, *ssaA* or *lytM*, found in its regulon, which has been shown to restore normal cell division under YycFG starvation (Delaune et al., 2011). By carrying out a genomic search for *ssaA* and *lytM*, it was found that the SE1457 genome possesses two *ssaA* genes, *ssaA1* (*serp1880*) and *ssaA2* (*serp2136*), but it does not contain *lytM*. The two *ssaA* genes share the same coding sequence with a different promoter sequence.

Two more plasmids, pMXssaA and pMXyycF-ssaA, were constructed and transformed into SE1457 to study the role of SsaA in *S. epidermidis*. The pMXssaA plasmid was used to express asRNA_*ssaA*_ and silence the two *ssaA* genes, while the pMXyycF-ssaA plasmid was used to constitutively overexpress SsaA and bring about the inducible expression of asRNA_*yycF*_. There was a significant decrease in bacterial growth due to asRNA_*ssaA*_, while the overexpression of SsaA partially prevented the growth inhibition effects of asRNA_*yycF*_. The pMXyycF-ssaA transformant entered log phase 2 h earlier than the SE1457::pMXyycF strain with ATc induction (Figure 3B).

The effect of *yycF* or *ssaA* silencing on bacterial cell morphology was observed with a transmission electron microscope. Silencing of either *yycF* or *ssaA* led to abnormal morphology, including cell enlargement, distorted shapes, and misplaced division septa. The disruption of the cell envelope resulted in cell death, with leaking of cytosol into the medium (Figure 3C). Meanwhile, none of the morphological changes were observed in the pMX6 transformant with ATc induction.

### Effect of asRNA*_*yycF*_* or asRNA*_*yycG*_* on biofilm formation

**Figure 4:**
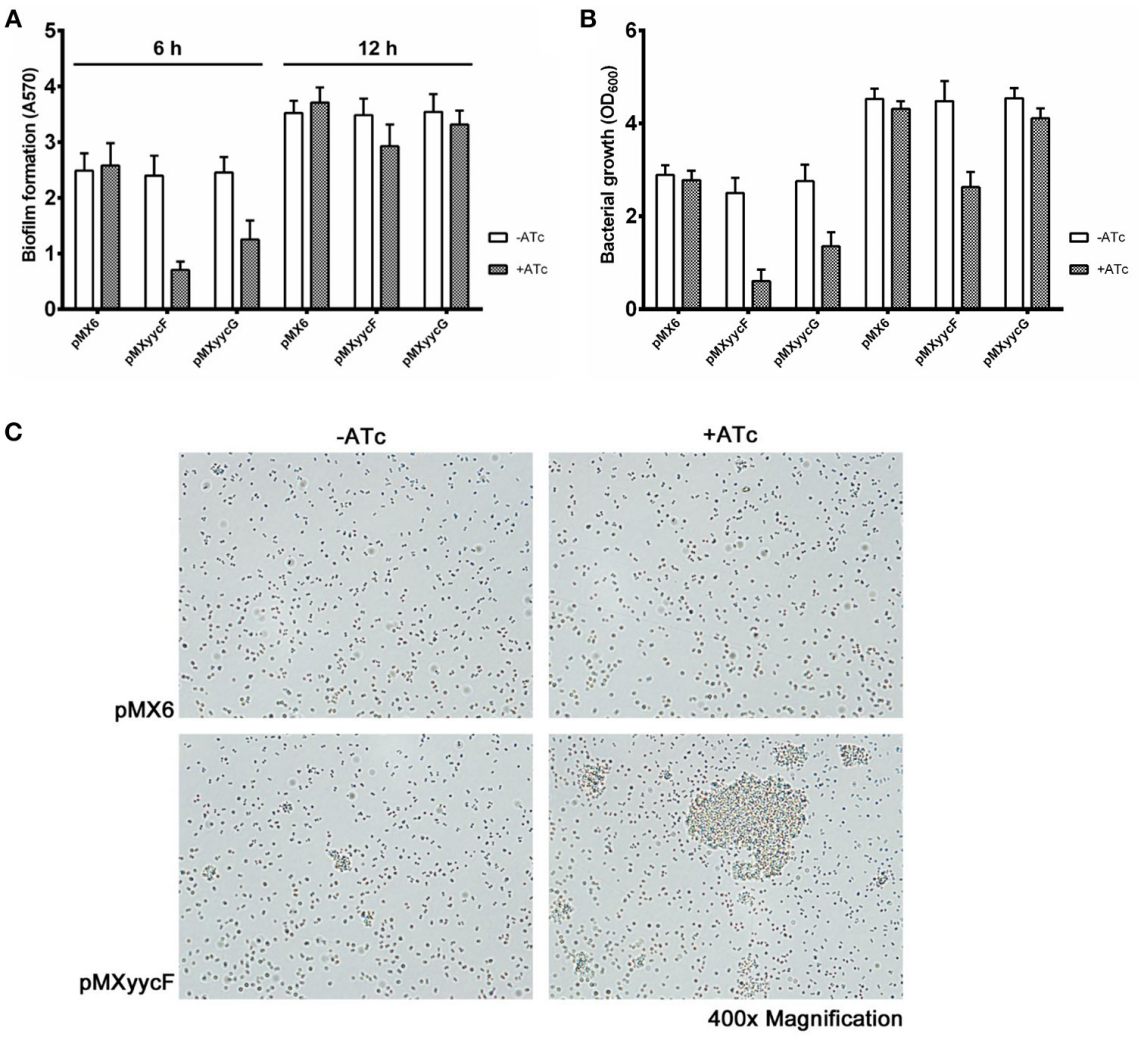
**Effects of ***yycF*** and asRNA_***yycG***_ on biofilm formation**. The strains were resuspended in TSB (1:200 dilution) and incubated in 96-well plates for 6 or 12 h. **(A)** Biofilm formation and **(B)** growth were measured by detecting the absorbance at 570 and 600 nm, respectively. **(C)** Primary attachment of the *S. epidermidis* 1457 strains to polystyrene surfaces. Overnight cultures grown to an OD_600_ of 0.6 were adjusted to an OD_600_ of 0.1 in PBS and inoculated into 6-well plates (2 ml/well). After 2 h at 37°C, the primary attachment at the bottom of the plates was observed under microscopy using a 40-fold objective lens.

We investigated the individual effects of asRNA_*yycF*_ and asRNA_*yycG*_ on biofilm formation *in vitro*. After ATc (250 ng/ml) induction for 6 h, asRNA_*yycF*_ and asRNA_*yycG*_ resulted in a decrease in biofilm formation of 68 and 50%, respectively, compared with the control without ATc (Figure 4A). Meanwhile, the inhibition of bacterial growth by asRNA_*yycF*_ (76%) was also greater than that caused by asRNA_*yycG*_ (51%) (Figure 4B). At 12 h, no significant inhibition of biofilm formation by asRNA_*yycF*_ or asRNA_*yycG*_ was observed but the growth inhibition by asRNA was still remarkable (Figure 4B).

Since *yycF* silencing showed more significant impacts on growth and biofilm formation than *yycG* silencing, we focused on asRNA_*yycF*_ in the subsequent analyses. Also, to overcome the interference to biofilm formation caused by growth inhibition, we detected the effects of asRNA_*yycF*_ on primary attachment, biofilm matrix production and biofilm gene expression with cultures at the same density (OD_600_ = 0.6), based on the consideration that cell density probably is a more important indicator of growth state than incubation time. By normalization of cell numbers, the effect of asRNA_*yycF*_ on primary attachment to polymer surfaces was assessed. After incubation in a 6-well polystyrene petri dish at 30°C for 30 min, SE1457 expressing asRNA_*yycF*_ formed more and much larger cell clusters compared with the control strains with or without ATc. The density of the attached bacterial cells in the areas without cell clusters was similar among all the strains (Figure 4C).

**Figure 5:**
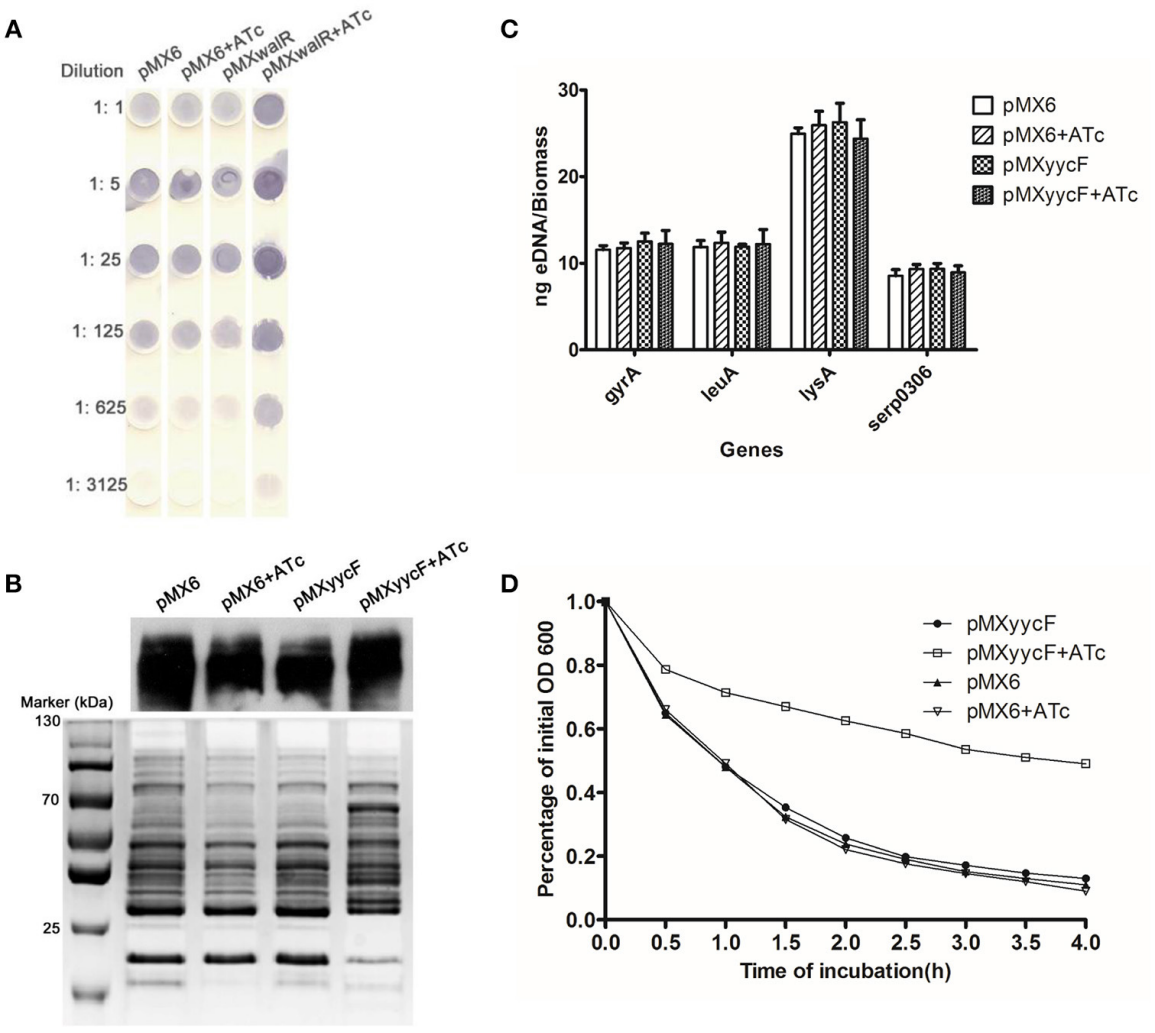
**Effects of asRNA_***yycF***_ on EPS production, Aap expression, and autolysis. (A)** Detection of PIA synthesis by *S. epidermidis* after silencing *yycF*. Serial dilutions of the PIA extractions detected using spot assays. The data represent one of three independent experiments. **(B)** Detection of Aap synthesis after silencing *yycF*. Aap expression was detected using western blotting with MAb25C11 (1 ng/mL). After separation of the proteins using 7% SDS-PAGE, the gel sections carrying high-molecular-weight proteins (>130 kDa) were excised for the western blot assay, and the remaining gel was stained using Coomassie brilliant blue as the endogenous control. **(C)** Extracellular DNA quantification. Extracellular DNA was isolated from the supernatants of each culture. Q-PCRs of four chromosomal loci were performed for eDNA quantification. **(D)** Detection of effects of asRNA_*yycF*_ on autolysis. Cultures grown to an OD_600_ of 0.6 were re-adjusted to an OD_600_ of 1. Autolysis induced by Triton X-100 at 30°C in the presence of 0.1% Triton X-100. The lysis percentage was calculated as follows: [(OD_t0_ − OD_tx_ /OD_t0_) × 100%]. Experiments were performed three times independently.

The influence of asRNA_*yycF*_ on EPS, including PIA, Aap, and eDNA, was explored. The effects of asRNA_*yycF*_ on PIA production was detected using a semi-quantitative dot-blot assay with a WGA-HRP conjugate. After the addition of 250 ng/ml ATc, the silencing of *yycF* in SE1457 resulted in a ~5-fold increase in PIA production compared with when there was no ATc induction and with the control plasmid (Figure 5A). The production of a major biofilm associated protein Aap that forms intracellular ligands was also detected. After silencing of *yycF*, no obvious change in Aap expression (Western blot, Figure 5B) was observed. No significant impact of asRNA_*yycF*_ on release of eDNA, an important factor that stabilizes the second structure of biofilms, was observed either (by qPCR, Figure 5C). The results combined indicate that YycFG mainly affects production of PIA.

The strain with induced asRNA_*yycF*_ exhibited a high level of resistance to Triton X-100-induced autolysis (~50% lysis was observed), while the OD_600_ of the control strains with or without ATc dropped to ~20% (Figure 5D).

**Figure 6:**
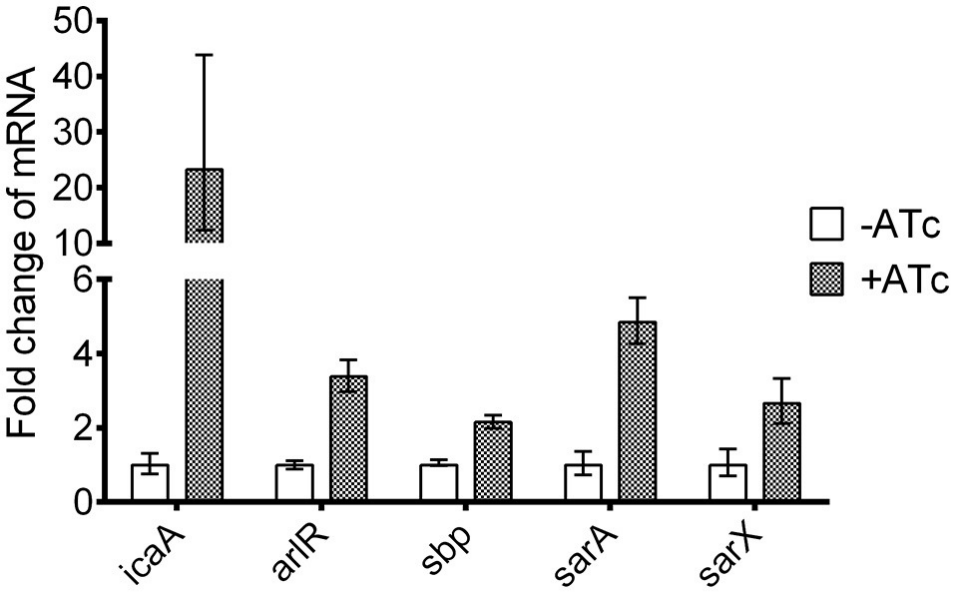
**Effects of asRNA_***yycF***_ on the expression of biofilm-related genes**. The expression of the genes in Table 2 was detected using qRT-PCR, with *gyrB* as an internal control. The experiment was carried out in triplicate and the expression ratios of the biofilm-related genes are represented as means with standard deviations.

We also used qRT-PCR to assess the effects of asRNA_*yycF*_ on the transcriptional levels of genes involved in biofilm formation (Table 2). The expression of *icaA, sbp, arlR, sarA*, and *sarX* clearly increased during asRNA_*yycF*_ induction (especially *icaA*) by more than 20-fold, while no significant change in the expression of other genes was observed (Figure 6).

**Table 2:** **Biofilm associated genes and detection**.

**Gene**	**qRT-PCR (ATc+/ATc–)**	**EMSA**	**Biological functions/pathways**	**References**
*icaA*	↑	+	PIA synthesis	Rohde et al., 2001
*icaR*	NC	+	Regulation of *icaADBC*	Jefferson et al., 2004
*sdrG*	NC	ND	Cell surface clumping factor	Hartford et al., 2001
*clpP*	NC	ND	Protease involved in biofilm formation	Michel et al., 2006)
*spx*	NC	−	Regulation of *icaADBC*	Wang et al., 2010
*ygs*	NC	−	Stress responder, regulator of PIA synthesis	Wang et al., 2011
*aap*	NC	+	Surface protein for accumulation	Conrady et al., 2008
*embp*	NC	−	Surface protein for intracellular adhesion	Christner et al., 2012
*sbp*	↑	ND	Scaffold protein for PIA and Aap	Decker et al., 2015
*agrA*	NC	−	Quorum sensing regulator	Lauderdale et al., 2009
*sigB*	NC	−	Regulation of PIA synthesis	Pane-Farre et al., 2006
*sarA*	↑	+	Regulation of PIA synthesis	Tormo et al., 2005
*sarX*	↑	+	Regulation of PIA synthesis	Rowe et al., 2011
*sarZ*	NC	−	Regulator of biofilm formation and virulence	Wang et al., 2008

*+, binding by YycF; −, no binding by YycF; NC, no change (cutoff = 2); ND, not detected*.

### Genome-based prediction of the *S. epidermidis* YycG/YycF regulon

*In silico* searches based on conserved motif pattern have been widely used among low-GC Gram positive bacterial species to provide information about the potential target genes that are directly regulated by YycF (Howell et al., 2003; Senadheera et al., 2012; Dhiman et al., 2014). To assess whether the pattern can be applied in *S. epidermidis*, the amino acid sequence of *S. epidermidis* YycF was compared with those of *B. subtilis* str. 168, *S. aureus* RN4220, and *S. mutans* UA159 using the Clustal X 2.0 program. The helix-turn-helix domain (180–217th amino acids) of the SE1457 YycF shares 100% identity with that of *S. aureus*, and it has a difference of one amino acid with the corresponding domain of *B. subtilis* (Supplementary Figure 3).

Based on the conserved pattern [5′-TGT(A/T)A(A/T/C)-N5-TGT(A/T)A(A/T/C)-3′], an *in silico* search of the *S. epidermidis* RP62A genome was carried out to predict the target genes of YycF, especially the biofilm-related target genes. We identified 28 potential binding sites of YycF, which were located in the promoter region (<400 bp upstream of each start codon) of various genes/operons (Table 3). The genes of the putative YycF regulon were divided into several groups, including metabolism (four genes/operons), protein production (three), phosphor transport (three), cell wall synthesis, and lysis (nine), as well as biofilm formation (two). The largest number of genes/operons (including both *ssaA* genes) were found to belong to the cell wall metabolism group, while only two genes (*arlR* and *sbp*) were found to belong to the biofilm formation group.

**Table 3:** **Genes potentially regulated by YycFG in ***S. epidermidis*****.

Predicted YycF binding sites^*^	**Gene**	**Gene symbol**	**Product**	**Biological functions/pathways**	**References**
−54 TGTAAATATTGTGTAAT →	*SERP_RS03290*	*qoxB*	Quinol oxidase, subunit II	Terminal oxidases involved in energy production	Winstedt and von Wachenfeldt, 2000
	*SERP_RS03285*	*qoxA*	Quinol oxidase, subunit I		
	*SERP_RS03280*	*qoxC*	Quinol oxidase, subunit III		
	*SERP_RS03275*	*qoxD*	Quinol oxidase, subunit IV		
−159 TGTAAATATATTGTTAT →	*SERP_RS02575*	*sufB*	FeS assembly protein	Iron-sulfur cluster biosynthesis	Fontecave and Ollagnier-de-Choudens, 2008
−175 TGTAAATCTAATGTTAA →	*SERP_RS02470*	*pgm*	Phosphoglycerate mutase	Glucose metabolism	Nukui et al., 2007
−317 TGTAATTATTATGTTAA →	*SERP_RS06410*		Chorismate mutase	Shikimate pathway	Kloosterman et al., 2003
−76 TGTAAAAAAACTGTTAA →	*SERP_RS11260*		Acetyl-CoA biotin carboxyl carrier	Fatty acid metabolism	
−105 TGTTAAACTTTTGTTAT →	*SERP_RS05210*	*rpsA*	30s ribosomal protein S1	Protein production	Agalarov et al., 2006
−47 GTAACAAAGCATTTACA←	*SERP_RS08380*	*tRNA-Leu-5*	tRNAs	Protein production	
	*SERP_RS08375*	*tRNA-Gly-5*			
−96 TTAACAAAAAATTTACA←	*SERP_RS04810*	*pstS*	Phosphate ABC transporters	Phosphate transport	Chan and Torriani, 1996
	*SERP_RS04805*	*pstC*			
	*SERP_RS04800*	*pstA*			
	*SERP_RS04795*	*pstB*			
	*SERP_RS04790*	*phoU*			
−229 TGTTAAGAATTTGTAAA →	*SERP_RS01725*	*pitR*	Transcription regulator	Zinc and phosphate transport	Mechler et al., 2015
−121 TTTACACAATTTTTACA←	*SERP_RS01730*	*pitA*	Transporter of Zn^2+^ in complex with Pi		
−43 TTAACAGTTTTTTTACA←	*SERP_RS11255*	*phnD*	Phosphonate transport system regulatory protein	Phosphonate transport	Gebhard and Cook, 2008
	*SERP_RS11250*				
	*SERP_RS11245*				
	*SERP_RS11240*				
−161 TTAACACTACCTTAACA←	*SERP_RS03100*	*murE*	UDP-N-acetylmuramoy-L-alanyl-D-glutamate-L-lysine ligase	Cell wall synthesis	Gardete et al., 2004; Zaher and Green, 2011
	*SERP_RS03105*				
	*SERP_RS03120*	*prfC*	peptide-chain-release factor 3	Protein production accuracy	
−226 ATTACAATAAGATTACA←	*SERP_RS02025*	*ltaS*	Glycerol phosphate lipoteichoic acid synthase	Lipoteichoic acid synthesis	Grundling and Schneewind, 2007b
−243 ATTACAAATGTATTACA←	*SERP_RS02885*	*oatA*	O-acetyltransferase	Teichoic acid modification	Bera et al., 2005
−344 TGTTAAGGTAGTGTTAA →	*SERP_RS03095*	*ltaA*	Diacylglycerol glucosyltransferase	Cell wall synthesis	Grundling and Schneewind, 2007a
	*SERP_RS03090*	*ypfP*		Lipoteichoic synthesis	
−112 TGTAATTGTAGTGTAAA →	*SERP_RS08520*	*sceD*	Lytic Transglycosylases	Cell wall hydrolase	Stapleton et al., 2007
−146 GTTACAAGATAATAACA←	*SERP_RS01735*		LysM domain protein	Cell wall hydrolase	
−77 ATTACATCAATATAACA←	*SERP_RS02225*		LysM domain autolysin	Cell wall hydrolase	
−269 TTTACATTCATGTAACA←	*SERP_RS09425*	*ssaA1*	CHAP domain autolysin	Cell wall hydrolase	Lang et al., 2000
−140 ATTACAAATCAATAACA←					
−252 ATTACAAGAATATAACA←	*SERP_RS10600*	*ssaA2*	CHAP domain autolysin	Cell wall hydrolase	
−146 ATTACAAAATGATAACA←					
−63 ATAACAAATCATTTACA←	*SERP_RS04480*	*arlR*	DNA-binding response regulator ArlR	Two-component system	Wu et al., 2014
	*SERP_RS04475*	*arlS*	Histidine kinase		
−287 TGTTACATGAATGTAAA →	*SERP_RS09420*				Schuller et al., 2012
	*SERP_RS09415*	*sarY*	AraC family transcriptional regulator	Transcription regulation	
	*SERP_RS09410*				
−69 TGTAATATTATTGTTAT →	*SERP_RS01525*	*sbp*	Scaffold protein	Biofilm formation	Decker et al., 2015
−248 TGTTATTATCTTGTAAC →	*SERP_RS01740*		Conserved hypothetical protein		
−63 TGTAACATTAATGTAAT →	*SERP_RS11965*		Conserved hypothetical protein		
−179 TGTAAAGTTGATGTTAT →	*SERP_RS01900*		Conserved hypothetical protein		

* *The numbers indicate the distance between the start codon of putative YycF target gene and the putative binding sites in the promoter region*.

### Binding of YycF to the predicted target genes

**Figure 7:**
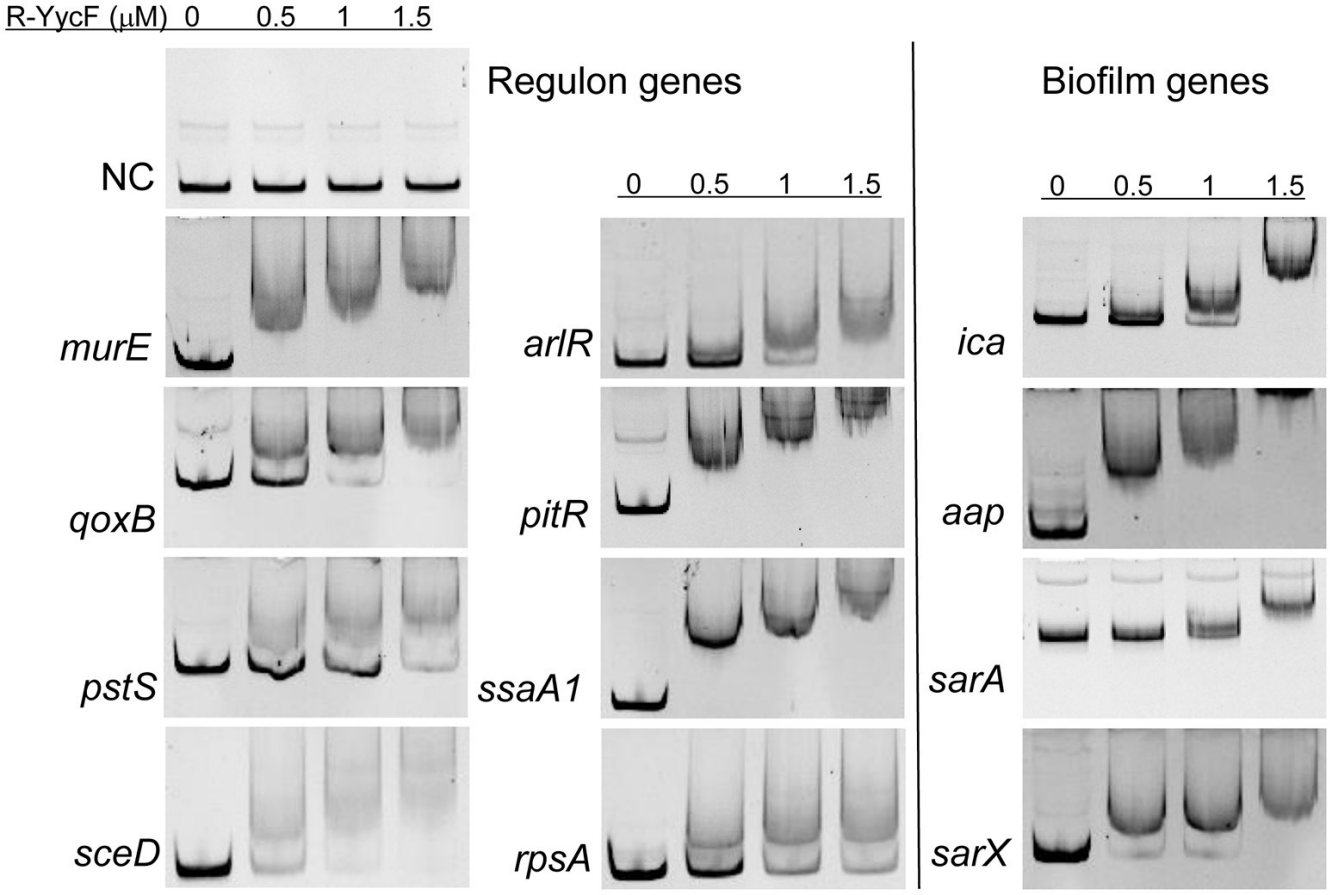
**Binding of r-YycF to the YycF regulon and biofilm-related genes**. Electrophoretic mobility shift assay using purified r-YycF with promoter regions of YycF target genes. DNA segments were amplified from the promoters of predicted YycF target genes and biofilm-related genes. For *ica*, all 164 nt between the coding sequence of *icaA* and *icaR* were used. For *sarX*, the promoter of serp3220, which is located upstream of sarX in the same operon, was amplified. A segment of the yycF coding sequence was used as a negative control.

**Figure 8:**
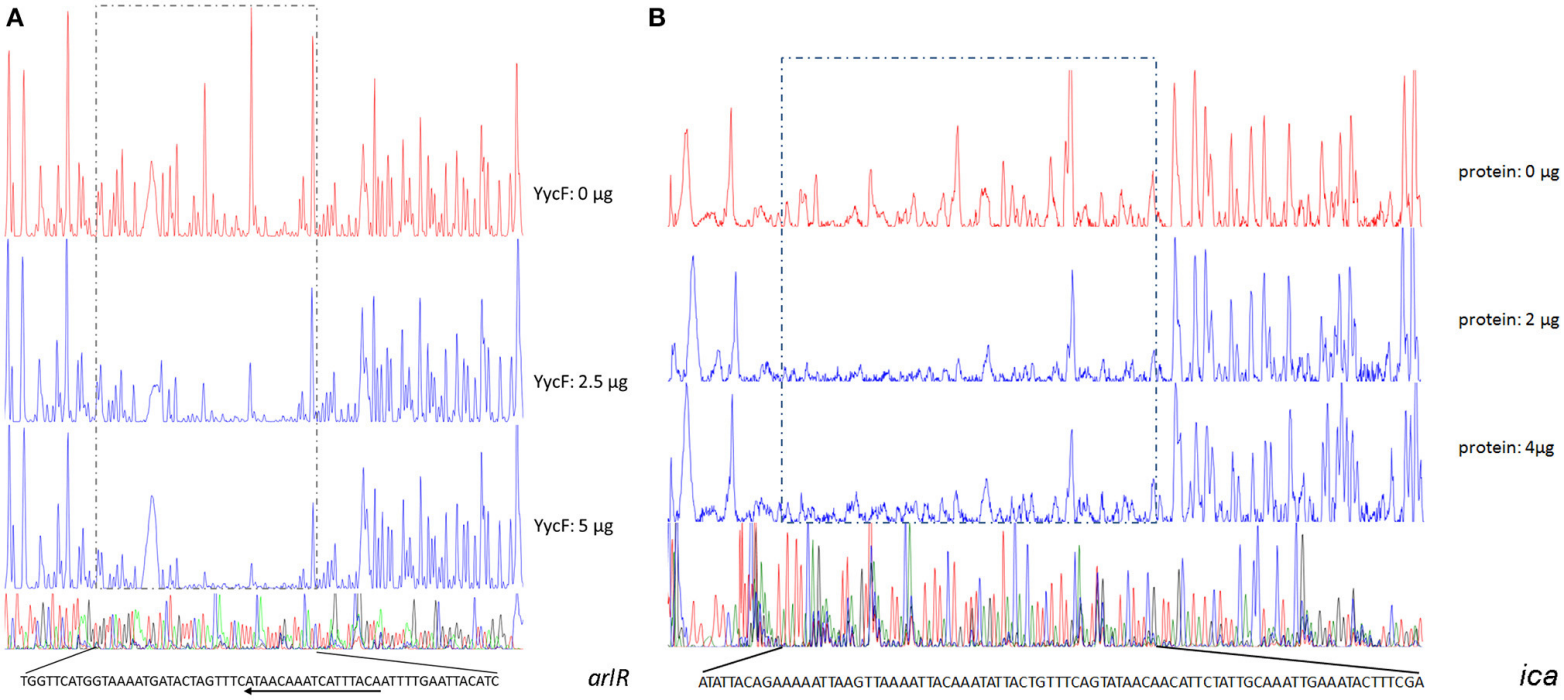
**Identification of the YycF-protected cis-elements in the promoter regions of (A)** *arlR* and **(B)** *ica* using DNase I footprinting assays. The regions protected by r-YycF are marked with frames of dashed lines. The DNA sequences of the protected regions are provided, and the sequences that are consistent with the previously reported YycF binding pattern are underlined. The arrows on these lines indicate the direction of the corresponding genes of the promoters.

To verify the predicted YycF target genes in *S. epidermidis*, a gel shift assay was performed. The recombinant YycF (r-YycF) was able to bind promoters of all eight selected genes: the r-YycF resulted in a mobility shift of the 188, 180, 167, 103, 159, 249, 232, and 150-bp fragments upstream of *murE, qoxB, pstS, sceD, arlR, pitR, ssaA1*, and *rpsA*, respectively, in a concentration-dependent manner. The negative control, a 125-bp DNA fragment of the *yycF* coding sequence, did not form a complex with r-YycF under the same conditions (Figure 7). The r-YycF protected region in the promoters of *arlR, qoxB*, and *pitR* was detected using the DNase I footprinting assay. A 60-nt protected region located upstream of the translational start site of *arlR* (−107 to −48 bp) was identified (Figure 8A). The 52-nt protected region in the promoter region of *qoxB* was located at −103 to −52 bp (Supplementary Figure 4A). Two separate protected regions (−282 to −196 bp, 87 nt; −152 to −114 bp, 39 nt) were identified in the *pitR* promoter region (Supplementary Figure 4B). The sequences of the protected region of the *arlR, qoxB*, and *pitR* promoters fit the consensus motif (Table 3).

To investigate whether the YycG/F TCS could bind the promoters of other genes in a motif-independent way, we further assessed the binding of r-YycF to promoters of the biofilm-related genes listed in Table 2. The r-YycF led to a mobility shift of the 83, 271, 322, and 361-bp fragments upstream of *ica, aap, sarA*, and *sarX*, respectively, in a concentration-dependent manner (Figure 7). The DNase I footprinting assay was used to identify an 83-bp r-YycF protected area in the region between *icaR* and *icaA* (Figure 8B).

**Figure 9:**
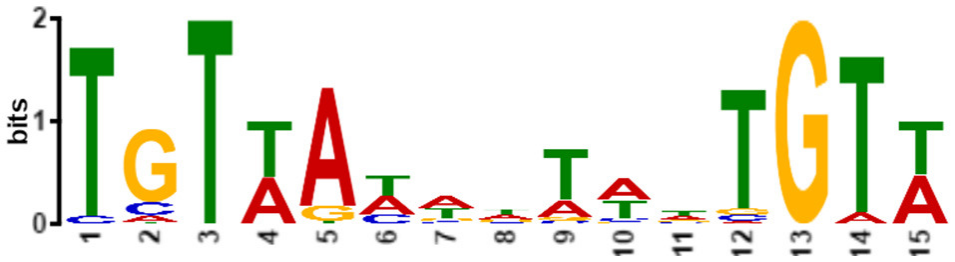
**A graphic depiction of an extended motif for YycF to recognize target genes in ***S. epidermidis*****. The conservative property of each base is indicated with heights of each letter.

Based on the high similarity of the YycF helix-turn-helix domain between *B. subtilis* and *S. epidermidis*, we gathered together previously reported atypical promoter sequences that YycF binds to in *B. subtilis* as well as those discovered in *S. epidermidis* in this study (Supplementary Table 1). A motif-based sequence analysis was performed to generate a new pattern with relaxed restrictions (Figure 9). By performing an *in silico* search of the *S. epidermidis* genome for the new pattern, more than 300 potential YycF binding sites were identified (Supplementary Table 2). The corresponding genes included genes that are involved in metabolism (*fmtC, tdk, gpmA*, and *glmU*), translation (*rbfA, rpsF, rrsD*, and *prfB*), and biofilm formation (*atlE, rsbU, ebh*, and *sarR*).

## Discussion

In the current study, we investigated the regulatory role of YycFG TCS in *S. epidermidis* biofilm formation by means of *in vitro* experiments and *in silico* techniques. With the results combined, we showed that YycFG TCS is a key regulator for *S. epidermidis* viability and negatively regulates *S. epidermidis* biofilm formation in an ica-dependent way.

Since YycFG TCS is essential for bacteria survival, we could not create knockout mutants of the *S. epidermis* genes. To study the regulatory functions of YycFG TCS in *S. aureus*, several methods have been used including site mutations (Fabret and Hoch, 1998), truncation of YycF (Gutu et al., 2010), and replacement of the promoter in the genome with an inducible promoter (Fukuchi et al., 2000; Dubrac and Msadek, 2004). In the present study, an asRNA silencing technique was applied. Our results showed that asRNA was highly effective for silencing genes, so it was able to reduce the *yycF* mRNA level by more than 99% at 6 h (Figure 2B). However, the efficacy decreases over time, as was reported by other researches (Ji et al., 2004; Stary et al., 2010). By detection of asRNA and mRNA of *yycF* or *yycG*, we showed that while the asRNA levels decreased from 6 to 12 h, the levels of target mRNAs increased (Figure 2). The result was consistent with the change of inhibitory effects of asRNA_*yycF*_ or asRNA_*yycG*_ on bacterial growth and biofilm formation (Figures 3A, 4A,B). However, the reason for the decrease of asRNA expression with time remains to be investigated in further study. In addition, the asRNAs acted specifically against the target mRNAs. The asRNA_*yycF*_ targeting the 5′ end of *yycF* (*yycF-5*) led to a decrease of over 99% in the *yycF-5* mRNA level (Figure 1), but it barely affected *yycF-3* or the three following genes (*yycG, yycH*, and *yycI*) (Supplementary Figure 5). The base pairing characteristic confers the asRNA technology the advantage to specifically silence the target gene without affecting the other genes in the same operon, and thus prevents the polar effects to the other genes in the same operon brought by promoter replacement (Fan et al., 2001).

As cell density affects biofilm formation, we explored the effects of asRNA_*yycF*_ when the OD_600_ of each bacterial culture reached 0.6. Our results suggested that YycF upregulates cell aggregation (Figure 4C), PIA formation (Figure 5A), bacterial autolysis (Figure 5D), and the expression of biofilm-related genes (*arlRS, icaA, sbp, sarA*, and *sarX*, as shown in Figure 6). No significant change in Aap production was observed. However, Aap-mediated cell aggregation may be enhanced by an elevated expression of Sbp, which increases the bridging of Aap B domains between bacterial cells (Decker et al., 2015). Although asRNA_*yycF*_ repressed autolysis, it did not affect eDNA release. The abnormal morphology of bacterial cells after asRNA_*yycF*_ silencing may be attributable to the repression of SsaA, and *ssaA* silenced by asRNA had a similar impact on the bacteria (Figure 3C).

After prediction and verification of the YycF regulon with the conserved motif, we assessed the effects of asRNA_*yycF*_ on transcriptional levels of all YycF regulon genes by qRT-PCR, The expression of most of the regulon genes was not affected, except that *rpsA* was up regulated for about 4-folds and *ssaA* down regulated for about 5-folds (Supplementary Figure 6). Meanwhile, based on the detection of asRNA effects on biofilm formation, we found that many other biofilm genes that did not appear in the predicted YycF regulon showed significant transcriptional change. The expression of *icaA, sbp, sarA*, and *sarX* (especially *icaA*) increased considerably (by more than 20-fold) after asRNA_*yycF*_ induction, while these genes were not identified from the YycF regulon (Figure 6). Furthermore, YycF is able to bind to the promoter regions of *ica, aap, sarA*, and *sarX* (Figures 8, 9B), proving that the recognition and regulation of target genes by YycF in *S. epidermidis* is not limited to the previously reported conserved pattern.

It has previously been reported that ArlRS positively regulates *S. epidermidis* biofilm formation in an *ica*-dependent manner (Wu et al., 2012). The mRNA level of *icaA* in Δ*arlS* was lower than that in the wild type strain (SE1457). When asRNA_*yycF*_ was introduced into the *arlS* knockout strain, induction of asRNA_*yycF*_ significantly increased the expression of *icaA* (Supplementary Figure 7), indicating that the YycFG TCS modulates biofilm formation mainly via the *ica*-dependent pathway, by regulation of *icaA* with other transcriptional regulators including ArlRS.

YycFG has been reported to regulate target genes in a conserved-motif-independent way in multiple bacterial species. In *B. subtilis*, several cell wall metabolism-associated genes (*yvcE1, yoeB2*, etc.) without the consensus recognition sequence in their promoter regions have been found to be directly controlled by YycF (Bisicchia et al., 2007). In a more recent study of *B. subtilis*, YycF-bound DNA was obtained using chromatin immunoprecipitation (ChIP), including many sites (*ggaA, lytE, dacA*, etc.) that do not fit the pattern either (Salzberg et al., 2012). More exceptions have been reported in *S. mutans* (*gtfB, smaA1, lysM, atlA*, etc., Senadheera et al., 2005; Stipp et al., 2013). To overcome the limitation of the consensus pattern, it can be modified based on *in vitro* experiments (Salzberg et al., 2012; Ayala et al., 2014). We performed the similar strategy to generate a new motif for prediction of more YycF regulon genes. The extended pattern provides more insights into YycF regulation in *S. epidermidis*. However, while the relaxation of certain sites allows so many more genes to be putative regulon genes, the binding ability of YycF to promoters of these genes requires further verification with gel shift assay.

*S. epidermidis* YycG histidine kinase inhibitors as well as *S. aureus* WalK/WalR inhibitors have potent antibacterial activities. In the present study, silencing *yycF* had more effect on the biological phenotype than silencing *yycG*, which indicates that the effect of asRNA that targets *yycF* may have some differences compared to the effect of asRNA that targets YycG. This indicates that YycG may function in other pathways through crosstalk with other TCSs, which requires further study.

**Figure 10:**
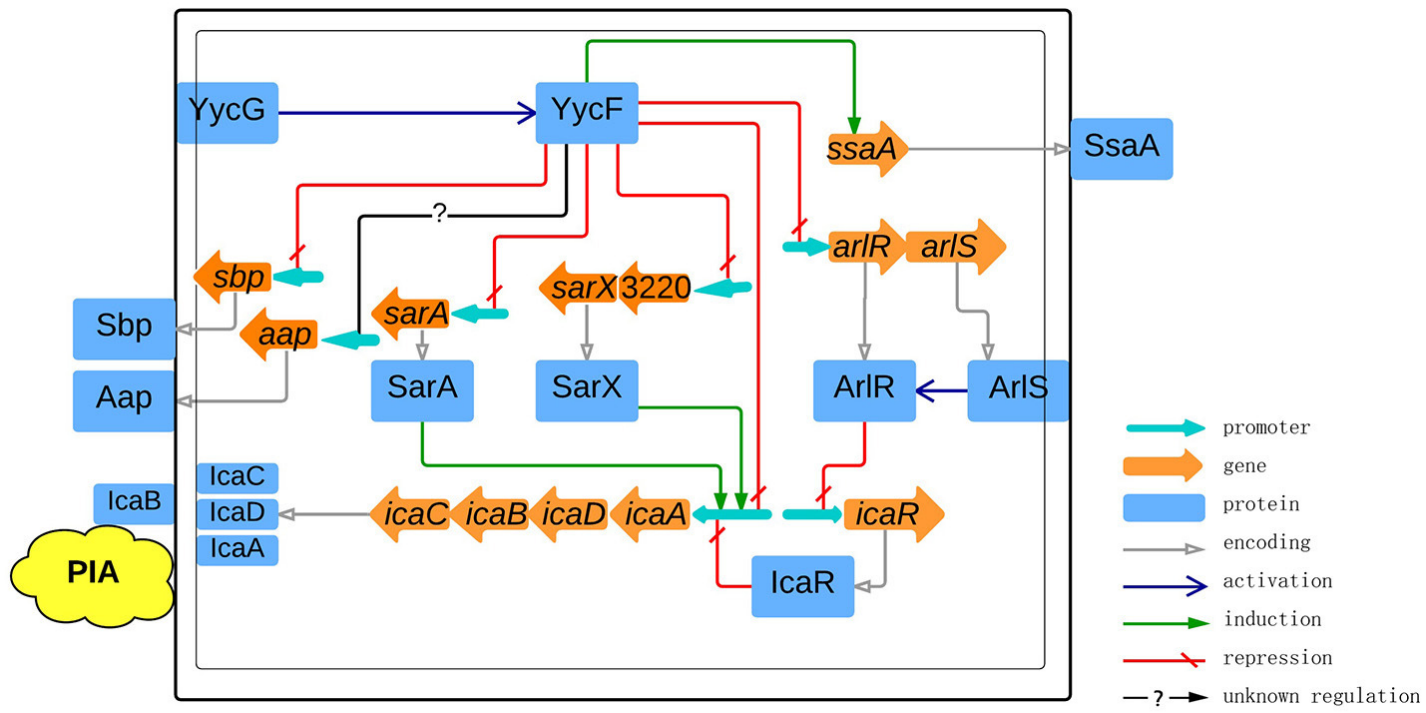
**Schematic representation of the roles of YycFG in PIA-dependent biofilm formation in ***S. epidermidis*****. The cytoplasmic cell membrane is represented by a thin border, in which YycG, ArlR, IcaA, IcaD, and icaC are embedded. The cell wall is represented by bold border, in which IcaB and PIA are embedded. The phosphorylation of YycF and ArlR by YycG and ArlS is signified by blue arrows. The genes are shown as brown arrows with an associated promoter (light blue arrows). The gray arrows represent the translation into protein. The induction and repression of transcription are represented by green arrows and red broken lines, respectively, while the question mark indicates that the regulation of transcription is indirect or that it occurs via an unclear mechanism.

In summary, by using a conserved and a modified motif pattern to search for the *S. epidermidis* YycF regulon, we found several YycFG target genes involved in energy production, translation, and cell wall metabolism, as well as biofilm formation. Based on confirmation of the regulation of biofilm-related genes by YycF, a model was established for the role of YycFG TCS in *S. epidermidis* biofilm formation (Figure 10). In addition to previous discoveries, we showed that YycF not only regulates biofilm-associated regulators such as *arlRS, sarA*, and *sarX*, but that it also binds to the promoters of *icaADBC* to directly modulate PIA production. The interaction of YycFG with other TCSs in *S. epidermidis* (by inter-regulation and crosstalk) warrants further investigation.

## Author contributions

DQ, YZ, FG, RB, and TX designed the work and revised the manuscript; TX, YW, and ZL completed all the experiments; TX and YW performed the statistically analysis and made the figures; TX, YW, and DQ wrote the manuscript.

### Conflict of interest statement

The authors declare that the research was conducted in the absence of any commercial or financial relationships that could be construed as a potential conflict of interest.

## Supplementary material

Supplementary Figure 1 **Representation of pMX6**. The relevant features are indicated with boxes (rep, replicon in *S. aureus*; cole1, replicon in *E. coli*), genes with arrows (*tetR*, TET repressor; *bla*, ampicillin resistant gene; *cat*, chloramphenicol resistance gene), promoters with triangles, and paired termini with rectangles.

Supplementary Figure 2 **Detection of asRNA_*yycG*_ and its effects on *yycG* mRNA**. Total RNA of cultures grown for 6 or 12 h was extracted and expression levels of asRNA **(A)** and mRNAs **(B)** were examined by qRT-PCR.

Supplementary Figure 3 **Alignment of amino acid sequences of YycF from *S. epidermidis* RP62A, *S. aureus* RN4220, and *B. subtilis* str. 168**. Shading indicates the conserved residues, and the two boxes indicate the DNA-binding winged helix-turn-helix domains. The amino acids at identity positions are marked with black backgrounds and the consensus positions are marked in gray.

Supplementary Figure 4 **DNase I footprinting assay of YycF binding property to promoters of *qoxB* (A)** and *pitR* **(B)**.

Supplementary Figure 5 **Impacts of asRNA_*yycF*_ on relative expression of genes in the *yyc* operon**. The *yycF* coding sequence were divided into two parts *yycF-5* and *yycF-3*, in which specific RT primers were located. The endogenous gene *gyrB* was used as an internal control. Similar results were obtained from three independent experiments.

Supplementary Figure 6 **Effects of asRNA_*yycF*_ on expression of regulon genes**. The selected genes in the list of YycF target genes were detected by qRT-PCR, using *gyrB* as an internal control. Genes showing significant changes (cutoff = 2-fold) on expression level were shown. Similar results were obtained from three independent experiments.

Supplementary Figure 7 **Impacts of asRNA_*yycF*_ on relative expression of *icaA* in SE1457 and *arlRS* mutant strain**. The endogenous gene *gyrB* was used as an internal control. Similar results were obtained from three independent experiments.

Supplementary Table 1 **Genes directly regulated by YycF with atypical promoter sequences**.

Supplementary Table 2 **Predicted regulon genes of YycF based on the new motif in *S. epidermidis***.
