# Supplementary figures and images for: Identification of Genes Controlled by the Essential YycFG Two-Component System Reveals a Role for Biofilm Modulation in Staphylococcus epidermidis
Source: Front Microbiol. 2017 Apr 26;8:724. doi: 10.3389/fmicb.2017.00724 (PMC5405149; doi:10.3389/fmicb.2017.00724)

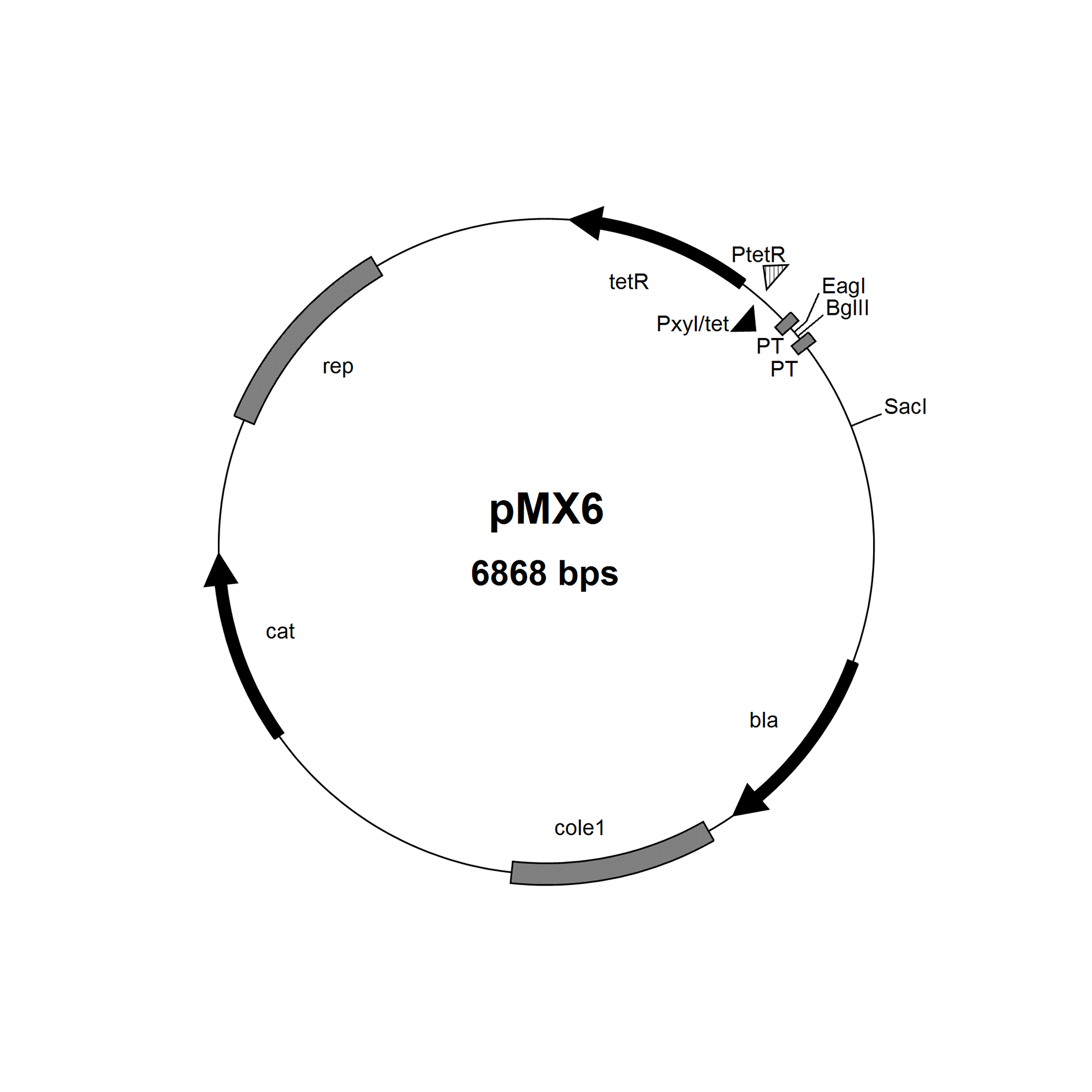

Supplement: Supplementary Figure 1 — Representation of pMX6. The relevant features are indicated with boxes (rep, replicon in S. aureus; cole1, replicon in E. coli), genes with arrows (tetR, TET repressor; bla, ampicillin resistant gene; cat, chloramphenicol resistance gene), promoters with triangles, and paired termini with rectangles. [file Image1.TIF]

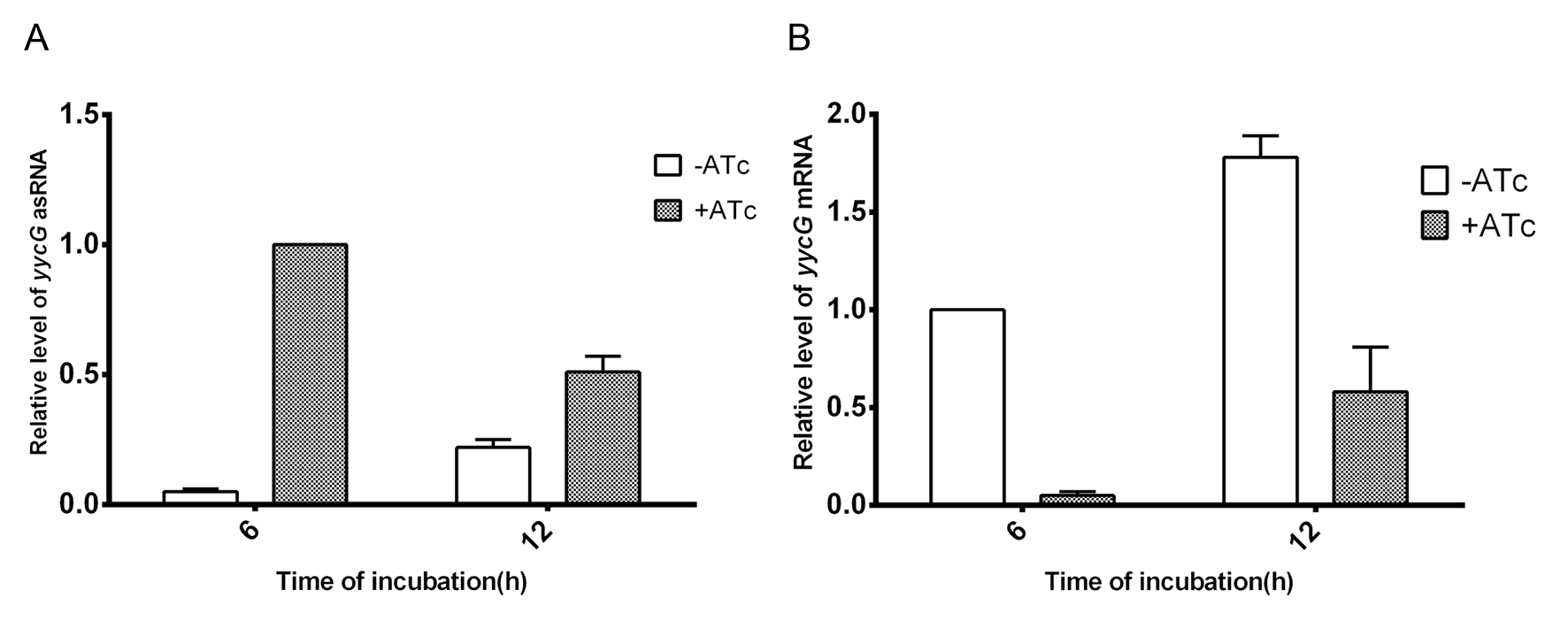

Supplement: Supplementary Figure 2 — Detection of asRNAyycG and its effects on yycG mRNA. Total RNA of cultures grown for 6 or 12 h was extracted and expression levels of asRNA (A) and mRNAs (B) were examined by qRT-PCR. [file Image2.TIF]

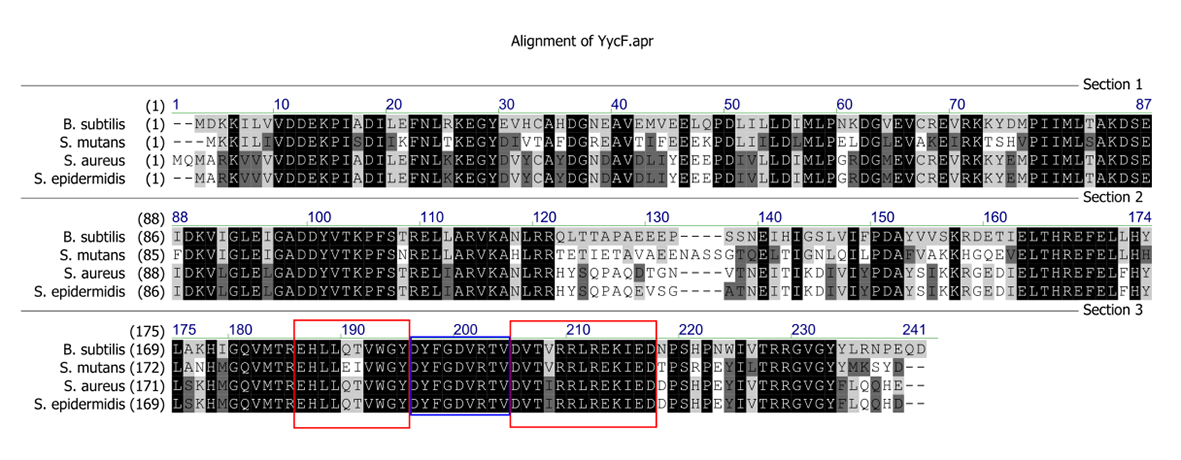

Supplement: Supplementary Figure 3 — Alignment of amino acid sequences of YycF from S. epidermidis RP62A, S. aureus RN4220, and B. subtilis str. 168. Shading indicates the conserved residues, and the two boxes indicate the DNA-binding winged helix-turn-helix domains. The amino acids at identity positions are marked with black backgrounds and the consensus positions are marked in gray. [file Image3.TIF]

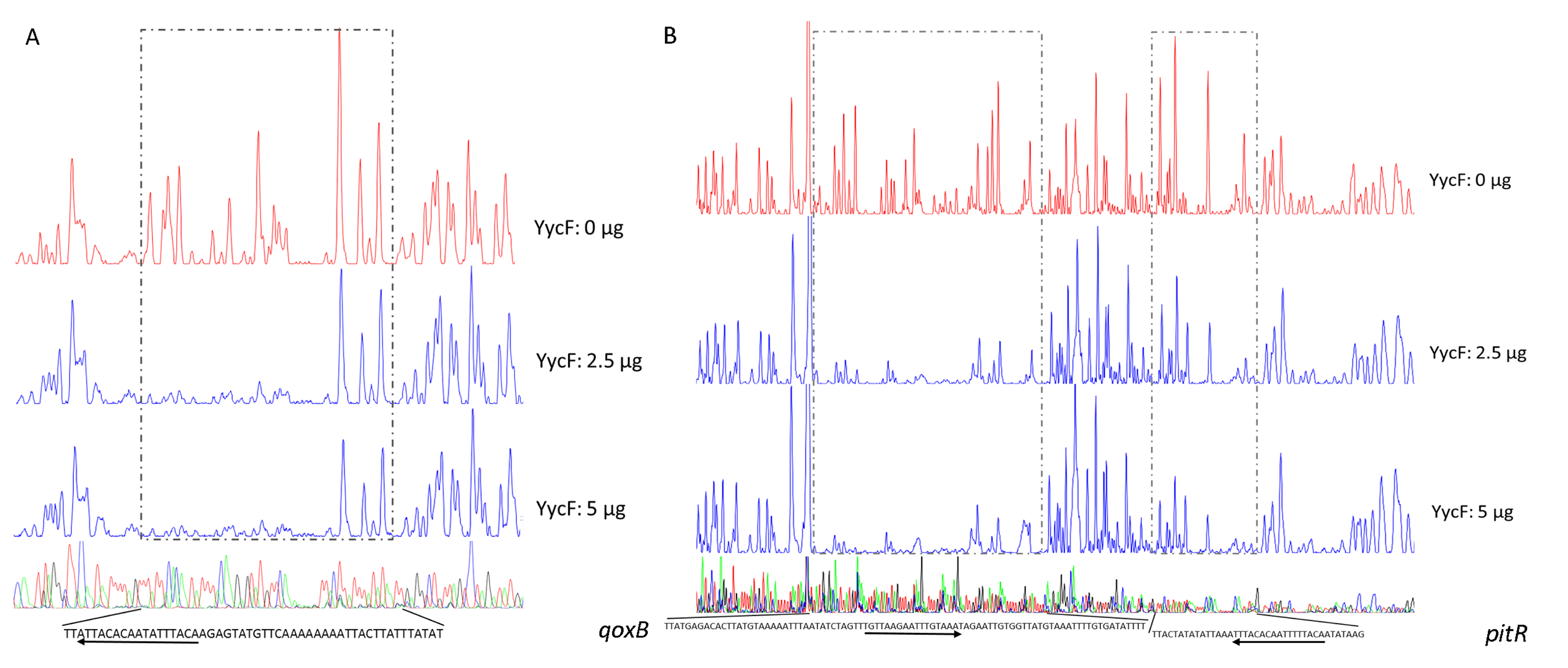

Supplement: Supplementary Figure 4 — DNase I footprinting assay of YycF binding property to promoters of qoxB (A) and pitR (B). [file Image4.TIF]

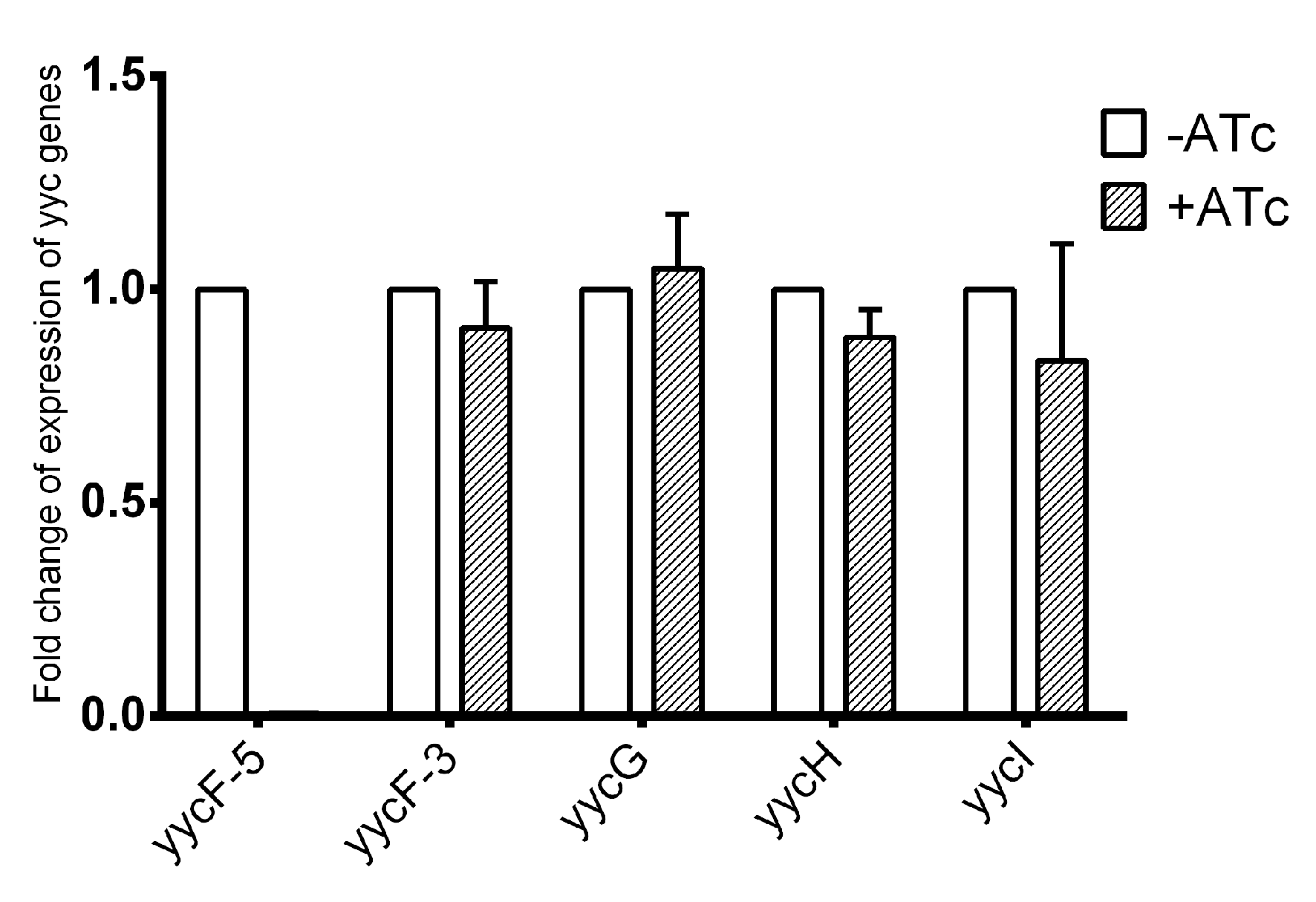

Supplement: Supplementary Figure 5 — Impacts of asRNAyycF on relative expression of genes in the yyc operon. The yycF coding sequence were divided into two parts yycF-5 and yycF-3, in which specific RT primers were located. The endogenous gene gyrB was used as an internal control. Similar results were obtained from three independent experiments. [file Image5.TIF]

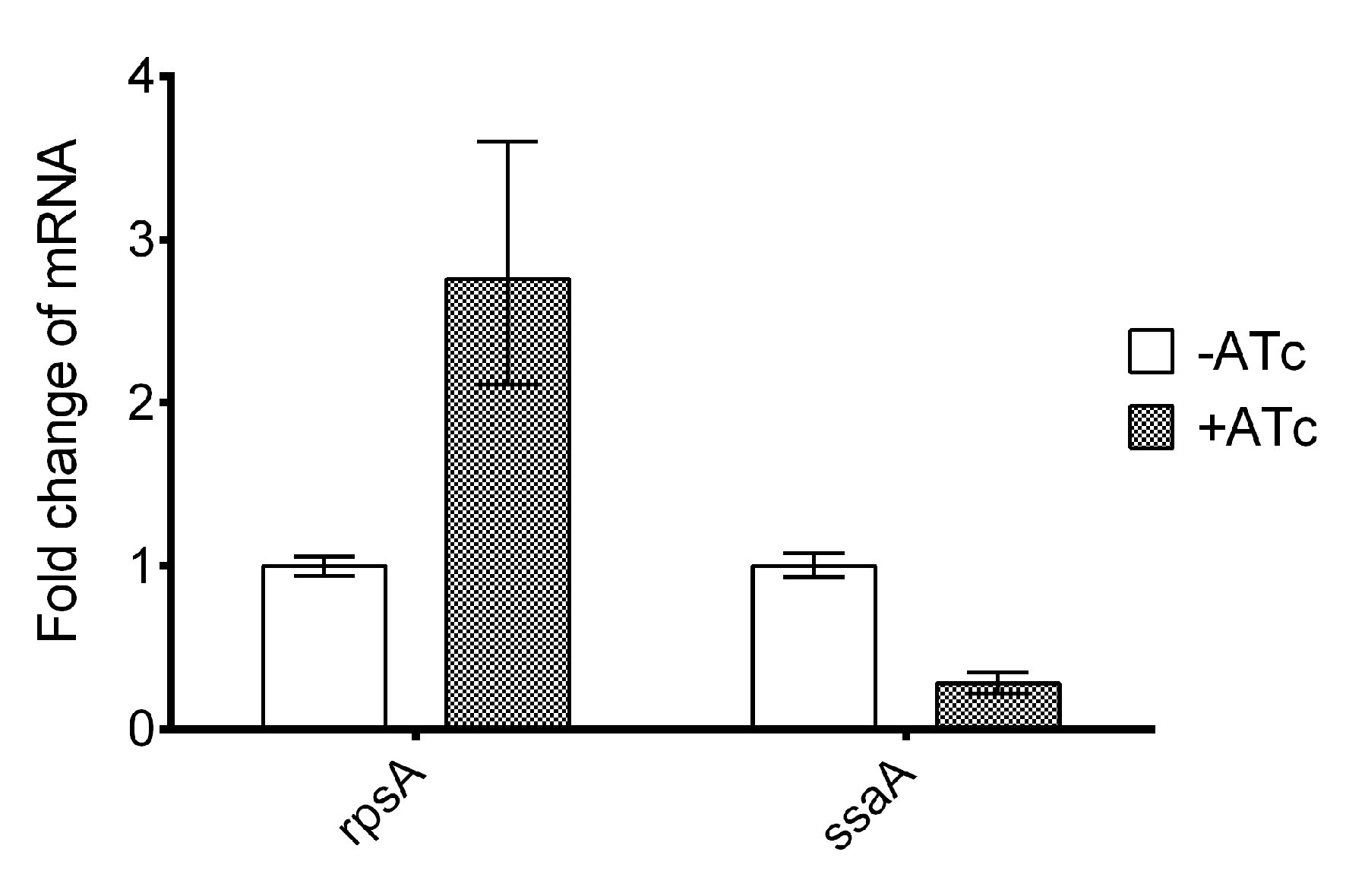

Supplement: Supplementary Figure 6 — Effects of asRNAyycF on expression of regulon genes. The selected genes in the list of YycF target genes were detected by qRT-PCR, using gyrB as an internal control. Genes showing significant changes (cutoff = 2-fold) on expression level were shown. Similar results were obtained from three independent experiments. [file Image6.TIF]

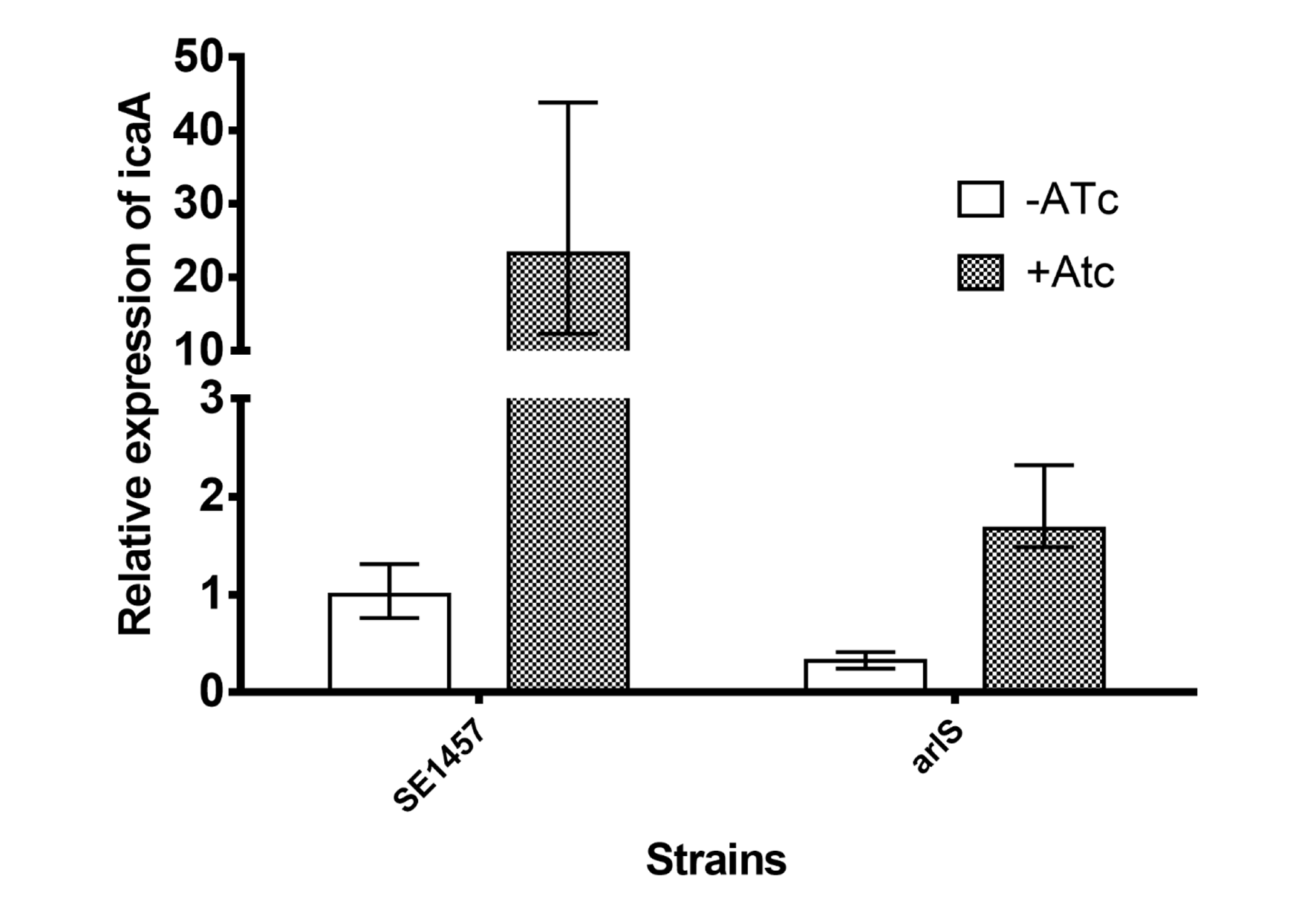

Supplement: Supplementary Figure 7 — Impacts of asRNAyycF on relative expression of icaA in SE1457 and arlRS mutant strain. The endogenous gene gyrB was used as an internal control. Similar results were obtained from three independent experiments. [file Image7.TIF]
