# Supplementary material for: Identification of Genes Controlled by the Essential YycFG Two-Component System Reveals a Role for Biofilm Modulation in Staphylococcus epidermidis
Source: Front Microbiol. 2017 Apr 26;8:724. doi: 10.3389/fmicb.2017.00724 (PMC5405149; doi:10.3389/fmicb.2017.00724)
Supplement: Supplementary Table 1 — Genes directly regulated by YycF with atypical promoter sequences. [file Table1.docx]

**Supplementary Table 1 Genes directly regulated by YycF with atypical promoter sequences**

| **Strain** | **Gene** | **Binding sequence 5’-3’** | **Detection** | **Reference** |
| --- | --- | --- | --- | --- |
| *B. subtilis* | *yvcE1* | ACTTGACATCTGTTCACAACATAAATGTAATAAAATTTATCGCTAATTTGTCGAAACTTGATGGTATAATGATTCTC | EMSA | (Bisicchia, Noone et al. 2007) |
|  | *ydjM* | AACTTTTTTGACTTTGTAACATTACTGTAAGGATATTGAAATAAAAAATAGCTGGTTGATCGTGTATAATCTTCCTA | EMSA | (Bisicchia, Noone et al. 2007) |
|  | *ydjM1* | AACTTTTTTGACTTTGTAACATTACCACAAGGATATTGAAATAAAAAATAGCTGGTTGATCGTGTATAATCTTCCTA | EMSA | (Bisicchia, Noone et al. 2007) |
|  | *ydjM2* | AACTTTTTTGACTTTGTAACATTACTGTAAGGATATTGAAATAAAAAATTTGATCGTGTATAATCTTCCTA | EMSA | (Bisicchia, Noone et al. 2007) |
|  | *ydjM3* | AACTTTTTTGACTTTGTAACATTACCACAAGGATATTGAAATAAAAAATTTGATCGTGTATAATCTTCCTA | EMSA | (Bisicchia, Noone et al. 2007) |
|  | *yoeB1* | CAAAAGTATTGTAATCTATCCGTAATTATTGTAACATTCACAACATAAGAGAAAGAGATTTTGAAGGAGAGAGCGATA | EMSA | (Bisicchia, Noone et al. 2007) |
|  | *yoeB2* | CAAAAGTATTGTAATCTATCAACAATTATTGTAACATTTGTAACATAAGAGAAAGAGATTTTGAAGGAGAGAGCGATA | EMSA | (Bisicchia, Noone et al. 2007) |
|  | *yjeA* | AAAAGGATTGACATGAACTTCTAAATCTCATAGTATTACAAATGTGAAATAAATGTTACTACAATGTTACAA | EMSA | (Bisicchia, Noone et al. 2007) |
|  | *yjeA1* | AAAAGGATTGACATGAACTTCTAAATCTCATAGTATTACAAATGTGAAATAAACACTACTACAACACTACAA | EMSA | (Bisicchia, Noone et al. 2007) |
|  | *lytE* | TAGAACGAATAATTAAGAAATTTGTCACATGAAGTCAAGACTATTTCTGATGGGAATCTATCCTTATAATAGAAATC | EMSA | (Bisicchia, Noone et al. 2007) |
|  | *phoA* | TAAACAAACATATCATGCAAAGACAGAGAGGTAAAGATTTTTCTGAAAAATGAATGCTTTACATCAAATAAGGCAAGATAACGAAAAGCGTTTTTTCATTTCCTTACAAGGCTTTACTTATTGTTTACATGATCAACAGCCGCATTTAACAAAGTTTCCCTAACATGATAAACGGAATACATTAAAGGAGGCATGAAAAAAATGAGTTTGTTTCAAAATATGAAATCAAAACTTCTGCCAATCGCCGCTGTTTCTGTCCTTACAGCTGGAATCTTTGCCGGAGCTGAGCTTCAGCAAACAGAAAAGGCCAGCGCCAAAAAACAAGACAAAGCTGAGATCAGAAATGTCATTGTGATGATAGGCGACGGCATGGGGACGCCTTACATAAGAGCCTACCGTTCCATGAAAAATAACGGTGACACACCGAATAACCCGAAGTTAACA | EMSA | (Bisicchia, Lioliou et al. 2010) |
|  | *phoPR* | AAATTTTCTTGTTCATGCTGTGCCTCCAGTATTATAATTCCCCGACATCCTATTTCTCTATTTTATGTCATCTTAACATGTTTCATGATTTTATTTTAGCGAAAGCAGGACATTCCGACAATTCGCCTTTTACATCATTTTTAAAAGAAATAAAGACGTTTATGATAGCGCTTTCATTTATAGTAAAAAGAGAAAGGCTGTATTAAGCAAGCCTTTCTCTCTTTTTATTAGGATAATACTTTCATGACATTTTTGACAGATTCAACTGATTTATTCAGCTGCGCTCTTTCATAGTCTGTCAGTTCAAGTTCAATGA | EMSA | (Bisicchia, Lioliou et al. 2010) |
|  | *cwlO* | TGAAACATAAATGTAATAAAATTTATCGCTAATTTGTCGAAACTTGATGGTATAAT | EMSA | (Salzberg, Powell et al. 2012) |
|  | *sigI* | TCTGAAAGACTCTGCTTAAAAGCAGAGTCTTTTTGTGTTTGAAGCGTTTTCATAAGCGAAATAAAATAACATTTTTATAACATCTTTGTTACAAAAAACACGCATAAAACCCCCTTAATTCTTTAGAAAGGCACGAAATCATGTATAGAACGTCAGAATGGTTTGTCATAATTGAGACATTGGAAATCAATATTTCTTTTTCTCCTTTGCGAATCCCTATCAAATTAGCTATCATTAATGAGTAGTTATAGGGAGGAACTGAG | EMSA | (Salzberg, Powell et al. 2012) |
| *Streptococcus mutans* | *gtfB* | CAATTAGACTGTTGTTTTTTTGTGGGATAGTTTTGTTTTTATCATGTTGTCATAATAAATAAAATAAAAAATTTGAATATTCTTTTTTATTTTTAAAGAAAAAAGAATTTTTGTTGCAAAAAGATTGTTTTATTATTAGAAAGGTGTTACAATTATAACGTTTTGAATAAAACAGTTTAAAATTTGGAGGTTCCTA | EMSA | (Senadheera, Guggenheim et al. 2005) |
|  | *sacB (ftf)* | CCACCCAAAATTTCCCTTTCTTATACATCTTTTTTCTAACTTTAGTTTCCATTAGCAAACCTCCTTTTTTATTATTGTTACATTATAATCCTAATTTATAACAAACACGATATCATTTATGTTAATTTTTAGAAACTTTATTTATGAAAAAATCGTTTTTTCATAAATTTCATCTCATTTTCATGACATAAAAACAAATTCACATATTAGATTAAG | EMSA | (Senadheera, Guggenheim et al. 2005) |
|  | *SMU.367* | CGTGGTCCTAGTCTTGTTATTTGgggaattcgagaaggacgcaaaacagcagagaaaattgatcaaaacctcagaatgatggttacagagtgagagtagaacaaaagagacaggactttcgtctcaggcttttctttgttatcaattagtaatagaaaaacttctttttcttaaaaaagttgaaataataattcggtatgatgttactattactaaaaggagaagttaagaataatcgttatgaaaaaacaatttttggaaaaagctgtgtttactgttgcggctacggcagcaacagttgttttagGAAATAAAATGGCTGATGCAG | DNase I Footprinting | (Stipp, Boisvert et al. 2013) |
|  | *smaA* | TGGAAGAGAAGATGTAACAATGAtttgtttaaatcttataaataagcatcaaccatttcttggtttggtgtttttttatatagtaaaaatttttcaaaaaatatattacgtaagtattgctaaatatttcttttgtgttacaatataggtgaaaaaagaaaatgaaggaagattatgaatcaaaaaatagtcgccatttcgtcattttacatgttcggtgctcattgattttcaaaggcagtatatcataatgataggagtgtgaaatttatgaaaagaattgatattaatcatcaagcacaacgtttttctattcgcaaatatgcatttggagctgCATCTGTTTTAATTGGCTGTGT | DNase I Footprinting | (Stipp, Boisvert et al. 2013) |
|  | *wapE* | GCTTCCGAACATTGATCTTATTaatattttaacaataaaaggttaggacaaaagtcctgcctttttcaaatatattaggcatagtagcttgaagtcagactgcttaactttctatttttgtcgatatgctcttaattgtattcattttttacagaaatgtaaaaaaaatgtgtccataataaaattaaatcatgataaactatattattattttaatgacattaaaataataataatttatttatgagaggaaaaaatggaacagaagatttttagcaaacgaaaaagtaagattgctgggCTTTGTGGAGCTATTTTAACGAC | DNase I Footprinting | (Stipp, Boisvert et al. 2013) |
|  | *SMU.2146c* | ATAATTTTATAGATATAGGTGCAAAATACTCAAAATTTTAATATTTTGGGTATTTTTTATTTAATTTAAATGCCAATGTAATATTTTTAATATAAAAATAAAATGAAAGTAATATTTAATCGATATTATAGAAATTGAGGTAATGAAAGGAATTAAGTTTT | DNase I Footprinting | (Stipp, Boisvert et al. 2013) |
|  | *lysM* | TGCAATTCCTGCAAAACTAACtttaattgttgttgttttgttttctaaaatatttttaatagacattggttactcctttaaacaaattatatatacatgatacagtagttatattacttgctttttagagtagtattaaaaatattacaattaagttgtgattagaaaaagtaaatgccttcttttccttttaagaaaagtaattccttatttatctgaaaaaaattgacttctaatagaatcgaagtattttctcatttttttaataaaaataaaataaaaccaagcaaacaaaTACCAACAATAGCCAAGCTGT | DNase I Footprinting | (Stipp, Boisvert et al. 2013) |
|  | *gbpB* | TTTCTCTTCTTTCAAAAACTTTTAGAAAGGATTTACCTATGAAAGGCGATGTTAAAGCAT  TTTAAATAAAAATGTAATAATGACGTAATATATGAAAAGGTTTTTTTTGATATAATTATA  AAGTCTTATCGAAAAAGGAGTTATATTTGTAATGAAAAAA | EMSA | (Ayala, Downey et al. 2014) |
|  | *bmsH* | TCCTCATTTCATCCATTTTGtctgttgcaactgacaccttcactatagcaagttttgtttaaaataaagtaacaaataattgcaagaaaataacatttttttaataaaagattaattagatattttatacccaaaaatgaTAGAAGCTCTTGAATTTTAT | EMSA | (Ayala, Downey et al. 2014) |
|  | *copY* | GTGAAGAGGCAGAAACTAAGgtttatcgcctctttttttgctcttttaaaaagctgataagtagggttatattttgtcaaaaaattaaaaatcagtttttgttgacaaatgtagacaaagaggttataatgtatctacaaatgtagatgaaaggagctcaAATGACATCTATTTCAAATG | EMSA | (Ayala, Downey et al. 2014) |
|  | *atlA* | TTACAATTATGAGGTTATTCctttttttgtttcaaaaaagtttctattttacaaaatttttctaattctcttgttattctgctaagggtcctttataatgataaggctaggagaaaatttGTGAATAAAAAACAGTCAGT | EMSA | (Ayala, Downey et al. 2014) |
|  | *relR* | AAAACGATGCCTTGCTGACTtcagttatttagtttgtttgtaacctcattatggcttggaacttctcataccatttggcaaaataagaattctttaatcttctatgttataatagaacaagcatgtaagaaggatgaattatgtcacaagaaacgatctatgggaaatatcaagcttattTGTCTCAGATTTTAGAGCAT | EMSA | (Ayala, Downey et al. 2014) |
| *S. epidermidis* | *ica* | ATATTACAGAAAAATTAAGTTAAAATTACAAATATTACTGTTTCAGTATAACAACATTCT  ATTGCAAATTGAAATACTTTCGA | EMSA & DNase I Footprinting | This study |
|  | *Aap* | GTTATCACTTACATAATCTATTCATACTAACTACGTTTTATAAATAGAGCTAGATGCCTATAGGTATCTAGCTTTTTTGTATTTAATATTCATTTACTTGTAATTACAATACCATCATAAAGCTGTCTCTTTTTAACATTATTATTGATTATTCAAATGCTTGTAGTTTTATAAGTCGCATTATTATTTTTAAATATTTTCACTATTGAATGGATTTTGATCTTCTTTATAAAAAATAAATAAAAGGTTTAAAGTTAAATAAAATTTAAAAAATAATAATTTATACAAAATGATATTTTTTTAATCATAAATAATATGTAAAGTATTGCGTTAAAATGTAAAAGAAGGTAACATTACATACTAGATACATAAAATATATGTATCTTACATTTAAAATTAAATACATGGGAGGTATAAT | EMSA | This study |
|  | *sarA* | AAAATGTTAGTAAAATTCTTTCCAAAAAGTATTCTTTTTAGTATAATTTTTATTAAATTAAGGAAAATTAGAAACAATCTTATTTTTTTTGAATATAGCAAATGCTACATTGCTAATTCAAGTATAAACTTTAAAAGATAATTTGTTTTATAAACACTTATTTGTTTACTTCTTAATTTTATTTAGTTATAATTAACTAAATAATATAGCATTAAATATATTCTTTAAATTTAAATTAATACTATATTATGACGGACACTTTCGTATTTTCATAAGAAAAATAGATATAAATTAATAAATTAGGGTATAGTATCCAAGTAATCTTAATTCAATAATATAATTAACTATCTTTAAATTTAAAAAGATGGGTTTTAAGATTTATGGATATGATATAAATAGGGAGGTTTCATTA | EMSA | This study |
|  | *sarX* | GTCAATTCTCACCAAGAGAAATGGGATTTAATTTGTTTATCAACTTATTAAATATCATCGAAGATATACTTAGTTTAGCAAATCGCTTTAAAAACTAAAGAGATTATGAATGAATTAAAAGATGTGATCGTGTTTCTCTATCAGAGAGTTTCTGGGTTTTTCATTTAATGTTGAACTCTCAACACGACACATCTTTTTTTATATCTAATAGAAAAGATGAAGTACAAAAATAAAAAAATGATTAAATATATCTTTTTCTTTTTGATTTATTTTTATATTGTGTTAGATTATTTTCTGATACTTGTGAAAATTTACATGAATTGATATGGGCATAAAAGACTGAGGGAAAG | EMSA | This study |

Ayala, E., J. S. Downey, L. Mashburn-Warren, D. B. Senadheera, D. G. Cvitkovitch and S. D. Goodman (2014). "A Biochemical Characterization of the DNA Binding Activity of the Response Regulator VicR from Streptococcus mutans." PLoS One **9**(9): e108027.

Bisicchia, P., E. Lioliou, D. Noone, L. I. Salzberg, E. Botella, S. Hubner and K. M. Devine (2010). "Peptidoglycan metabolism is controlled by the WalRK (YycFG) and PhoPR two-component systems in phosphate limited Bacillus subtilis cells." Mol Microbiol.

Bisicchia, P., D. Noone, E. Lioliou, A. Howell, S. Quigley, T. Jensen, H. Jarmer and K. M. Devine (2007). "The essential YycFG two-component system controls cell wall metabolism in Bacillus subtilis." Mol Microbiol **65**(1): 180-200.

Salzberg, L. I., L. Powell, K. Hokamp, E. Botella, D. Noone and K. M. Devine (2012). "The WalRK (YycFG) and sigma(I) RsgI regulators cooperate to control CwlO and LytE expression in exponentially growing and stressed Bacillus subtilis cells." Mol Microbiol.

Senadheera, M. D., B. Guggenheim, G. A. Spatafora, Y. C. Huang, J. Choi, D. C. Hung, J. S. Treglown, S. D. Goodman, R. P. Ellen and D. G. Cvitkovitch (2005). "A VicRK signal transduction system in Streptococcus mutans affects gtfBCD, gbpB, and ftf expression, biofilm formation, and genetic competence development." J Bacteriol **187**(12): 4064-4076.

Stipp, R. N., H. Boisvert, D. J. Smith, J. F. Höfling, M. J. Duncan and R. O. Mattos-Graner (2013). "CovR and VicRK Regulate Cell Surface Biogenesis Genes Required for Biofilm Formation in <italic>Streptococcus mutans</italic>." PLoS ONE **8**(3): e58271.
