# Supplementary material for: Identification of Genes Controlled by the Essential YycFG Two-Component System Reveals a Role for Biofilm Modulation in Staphylococcus epidermidis
Source: Front Microbiol. 2017 Apr 26;8:724. doi: 10.3389/fmicb.2017.00724 (PMC5405149; doi:10.3389/fmicb.2017.00724)
Supplement: Supplementary Table 2 — Predicted regulon genes of YycF based on the new motif in S. epidermidis. [file Table2.docx]

**Supplementary Table 2 Predicted regulon genes of YycF based on the new motif in *S. epidermidis***

| **Position** | **Gene** | **Gene name** | **Product** |
| --- | --- | --- | --- |
| -119 | SERP_Se16SD | *rrsD* | 16S ribosomal RNA |
| -338 | SERP_Se16SD | *rrsD* | 16S ribosomal RNA |
| -221 | SERP_Se16SF | *rrsF* | 16S ribosomal RNA |
| -241 | SERP_Se16SF | *rrsF* | 16S ribosomal RNA |
| -159 | SERP_Se23SD | *rrlD* | 23S ribosomal RNA |
| -33 | SERP0003 | *mnmE* | tRNA modification GTPase TrmE |
| -43 | SERP0004 | *gidA* | tRNA uridine 5-carboxymethylaminomethyl |
| -369 | SERP0044 | *rpsF* | 30S ribosomal protein S6 |
| -322 | SERP0044 | *rpsF* | 30S ribosomal protein S6 |
| -305 | SERP0044 | *rpsF* | 30S ribosomal protein S6 |
| -234 | SERP0044 | *rpsF* | 30S ribosomal protein S6 |
| -28 | SERP0044 | *rpsF* | 30S ribosomal protein S6 |
| -157 | SERP0060 | *ahpC* | alkyl hydroperoxide reductase |
| -323 | SERP0060 | *ahpC* | alkyl hydroperoxide reductase |
| -301 | SERP0067 | *xpt* | xanthine phosphoribosyltransferase |
| -254 | SERP0067 | *xpt* | xanthine phosphoribosyltransferase |
| -286 | SERP0084 | *nuoF* | NADH dehydrogenase subunit 5 |
| -164 | SERP0084 | *nuoF* | NADH dehydrogenase subunit 5 |
| -121 | SERP0128 | *metS* | methionyl-tRNA synthetase |
| -309 | SERP0133 | *ipk* | 4-diphosphocytidyl-2-C-methyl-D-erythritol |
| -123 | SERP0133 | *ipk* | 4-diphosphocytidyl-2-C-methyl-D-erythritol |
| -22 | SERP0133 | *ipk* | 4-diphosphocytidyl-2-C-methyl-D-erythritol |
| -314 | SERP0137 | *glmU* | bifunctional N-acetylglucosamine-1-phosphate |
| -197 | SERP0137 | *glmU* | bifunctional N-acetylglucosamine-1-phosphate |
| -169 | SERP0137 | *glmU* | bifunctional N-acetylglucosamine-1-phosphate |
| -164 | SERP0137 | *glmU* | bifunctional N-acetylglucosamine-1-phosphate |
| -94 | SERP0137 | *glmU* | bifunctional N-acetylglucosamine-1-phosphate |
| -30 | SERP0137 | *glmU* | bifunctional N-acetylglucosamine-1-phosphate |
| -77 | SERP0138 | *prsA* | ribose-phosphate pyrophosphokinase |
| -117 | SERP0139 | *rplY* | 50S ribosomal protein L25 |
| -145 | SERP0140 | *pth* | peptidyl-tRNA hydrolase |
| -16 | SERP0140 | *pth* | peptidyl-tRNA hydrolase |
| -22 | SERP0152 | *cysK* | cysteine synthase |
| -128 | SERP0153 | *folP* | dihydropteroate synthase |
| -316 | SERP0156 | *lysS* | lysyl-tRNA synthetase |
| -265 | SERP0156 | *lysS* | lysyl-tRNA synthetase |
| -104 | SERP0166 | *radA* | DNA repair protein RadA |
| -26 | SERP0175 | *rpmG* | 50S ribosomal protein L33 |
| -12 | SERP0178 | *rplK* | 50S ribosomal protein L11 |
| -116 | SERP0179 | *rplA* | 50S ribosomal protein L1 |
| -38 | SERP0188 | *fusA* | elongation factor G |
| -47 | SERP0211 | *arsR-1* | arsenical resistance operon repressor |
| -80 | SERP0215 | *nagB* | glucosamine-6-phosphate isomerase |
| -47 | SERP0215 | *nagB* | glucosamine-6-phosphate isomerase |
| -18 | SERP0215 | *nagB* | glucosamine-6-phosphate isomerase |
| -20 | SERP0236 | *eutD* | phosphotransacetylase |
| -233 | SERP0257 | *adhA* | alcohol dehydrogenase |
| -133 | SERP0257 | *adhA* | alcohol dehydrogenase |
| -348 | SERP0274 | *sarA* | accessory regulator A |
| -40 | SERP0300 | *tagD* | glycerol-3-phosphate cytidylyltransferase |
| -326 | SERP0303 | *abcA* | ABC transporter permease/ATP-binding protein |
| -6 | SERP0303 | *abcA* | ABC transporter permease/ATP-binding protein |
| -75 | SERP0360 | *nagA* | N-acetylglucosamine-6-phosphate deacetylase |
| -186 | SERP0374 | *pabA* | para-aminobenzoate synthase, glutamine |
| -90 | SERP0374 | *pabA* | para-aminobenzoate synthase, glutamine |
| -32 | SERP0390 | *bmrU* | bmrU protein |
| -140 | SERP0390 | *bmrU* | bmrU protein |
| -32 | SERP0405 | *murB* | UDP-N-acetylenolpyruvoylglucosamine reductase |
| -167 | SERP0420 | *azi* | preprotein translocase subunit SecA |
| -63 | SERP0420 | *azi* | preprotein translocase subunit SecA |
| -118 | SERP0421 | *prfB* | peptide chain release factor 2 |
| -249 | SERP0421 | *prfB* | peptide chain release factor 2 |
| -252 | SERP0421 | *prfB* | peptide chain release factor 2 |
| -292 | SERP0421 | *prfB* | peptide chain release factor 2 |
| -76 | SERP0428 | *hprK* | HPr kinase/phosphorylase |
| -63 | SERP0428 | *hprK* | HPr kinase/phosphorylase |
| -7 | SERP0436 | *clpP* | ATP-dependent Clp protease proteolytic subunit |
| -13 | SERP0441 | *gapR* | gap transcriptional regulator |
| -27 | SERP0485 | *gcvH* | glycine cleavage system protein H |
| -173 | SERP0496 | *sufC* | FeS assembly ATPase SufC |
| -86 | SERP0496 | *sufC* | FeS assembly ATPase SufC |
| -162 | SERP0500 | *sufB* | FeS assembly protein SufB |
| -154 | SERP0500 | *sufB* | FeS assembly protein SufB |
| -80 | SERP0546 | *gluD* | glutamate dehydrogenase, NAD-specific |
| -49 | SERP0546 | *gluD* | glutamate dehydrogenase, NAD-specific |
| -41 | SERP0546 | *gluD* | glutamate dehydrogenase, NAD-specific |
| -49 | SERP0549 | *argG* | argininosuccinate synthase |
| -71 | SERP0549 | *argG* | argininosuccinate synthase |
| -284 | SERP0550 | *pgi* | glucose-6-phosphate isomerase |
| -262 | SERP0550 | *pgi* | glucose-6-phosphate isomerase |
| -96 | SERP0567 | *fabH* | 3-oxoacyl-ACP synthase |
| -221 | SERP0570 | *oppB* | oligopeptide ABC transporter permease |
| -99 | SERP0575 | *trpS* | tryptophanyl-tRNA synthetase |
| -178 | SERP0576 | *yjbD* | Spx family transcriptional regulator |
| -103 | SERP0606 | *ypfP* | diacylglycerol glucosyltransferase |
| -346 | SERP0606 | *ypfP* | diacylglycerol glucosyltransferase |
| -161 | SERP0607 | *murE* | UDP-N-acetylmuramoylalanyl-D-glutamate--L-lysine |
| -105 | SERP0607 | *murE* | UDP-N-acetylmuramoylalanyl-D-glutamate--L-lysine |
| -132 | SERP0627 | *menA* | 1,4-dihydroxy-2-naphthoate |
| -74 | SERP0636 | *atlE* | bifunctional autolysin |
| -163 | SERP0636 | *atlE* | bifunctional autolysin |
| -56 | SERP0646 | *qoxB* | quinol oxidase, subunit II |
| -364 | SERP0646 | *qoxB* | quinol oxidase, subunit II |
| -49 | SERP0669 | *ptsH* | phosphocarrier protein HPr |
| -30 | SERP0669 | *ptsH* | phosphocarrier protein HPr |
| -145 | SERP0704 | *pyc* | pyruvate carboxylase |
| -42 | SERP0705 | *ctaA* | cytochrome oxidase assembly protein |
| -245 | SERP0706 | *ctaB* | protoheme IX farnesyltransferase |
| -295 | SERP0721 | *pheS* | phenylalanyl-tRNA synthetase subunit alpha |
| -216 | SERP0721 | *pheS* | phenylalanyl-tRNA synthetase subunit alpha |
| -145 | SERP0721 | *pheS* | phenylalanyl-tRNA synthetase subunit alpha |
| -135 | SERP0728 | *trxA* | thioredoxin |
| -145 | SERP0756 | *ylmH* | hypothetical protein |
| -31 | SERP0778 | *coaBC* | phosphopantothenoylcysteine |
| -303 | SERP0790 | *rpmB* | 50S ribosomal protein L28 |
| -176 | SERP0793 | *recG* | ATP-dependent DNA helicase RecG |
| -124 | SERP0793 | *recG* | ATP-dependent DNA helicase RecG |
| -6 | SERP0799 | *rnc* | ribonuclease III |
| -70 | SERP0818 | *xerC* | tyrosine recombinase XerC |
| -27 | SERP0818 | *xerC* | tyrosine recombinase XerC |
| -17 | SERP0820 | *hslU* | ATP-dependent protease ATP-binding subunit HslU |
| -103 | SERP0831 | *polC* | DNA polymerase III PolC |
| -103 | SERP0837 | *rbfA* | ribosome-binding factor A |
| -95 | SERP0837 | *rbfA* | ribosome-binding factor A |
| -261 | SERP0860 | *miaB* | (dimethylallyl)adenosine tRNA |
| -216 | SERP0860 | *miaB* | (dimethylallyl)adenosine tRNA |
| -48 | SERP0863 | *hexA* | DNA mismatch repair protein MutS |
| -43 | SERP0868 | *glpD* | aerobic glycerol-3-phosphate dehydrogenase |
| -354 | SERP0875 | *glnR* | glutamine synthetase repressor |
| -184 | SERP0875 | *glnR* | glutamine synthetase repressor |
| -104 | SERP0875 | *glnR* | glutamine synthetase repressor |
| -55 | SERP0904 | *rpmG* | 50S ribosomal protein L33 |
| -25 | SERP0905 | *rpsN* | 30S ribosomal protein S14 |
| -23 | SERP0905 | *rpsN* | 30S ribosomal protein S14 |
| -118 | SERP0909 | *lexA* | LexA repressor |
| -394 | SERP0909 | *lexA* | LexA repressor |
| -75 | SERP0912 | *tkt* | transketolase |
| -71 | SERP0917 | *sbcD* | exonuclease SbcD |
| -5 | SERP0917 | *sbcD* | exonuclease SbcD |
| -67 | SERP0921 | *acnA* | aconitate hydratase |
| -182 | SERP0925 | *parE* | DNA topoisomerase IV subunit B |
| -219 | SERP0930 | *fmtC* | fmtC protein |
| -216 | SERP0930 | *fmtC* | fmtC protein |
| -91 | SERP0930 | *fmtC* | fmtC protein |
| -88 | SERP0930 | *fmtC* | fmtC protein |
| -80 | SERP0931 | *msrA-1* | methionine sulfoxide reductase A |
| -115 | SERP0934 | *dmpI* | 4-oxalocrotonate tautomerase |
| -25 | SERP0936 | *tyrA* | prephenate dehydrogenase |
| -55 | SERP0936 | *tyrA* | prephenate dehydrogenase |
| -92 | SERP0937 | *trpE* | anthranilate synthase component I |
| -311 | SERP0963 | *lysC* | aspartate kinase |
| -261 | SERP0963 | *lysC* | aspartate kinase |
| -92 | SERP0977 | *brnQ-1* | branched-chain amino acid ABC transporter |
| -367 | SERP0986 | *sucA* | 2-oxoglutarate dehydrogenase E1 |
| -63 | SERP0989 | *arlR* | DNA-binding response regulator ArlR |
| -170 | SERP1003 | *thyA* | thymidylate synthase |
| -243 | SERP1003 | *thyA* | thymidylate synthase |
| -139 | SERP1011 | *ebh* | cell wall associated fibronectin-binding |
| -154 | SERP1011 | *ebh* | cell wall associated fibronectin-binding |
| -296 | SERP1019 | *recU* | Holliday junction-specific endonuclease |
| -64 | SERP1036 | *aroC* | chorismate synthase |
| -93 | SERP1041 | *hup* | DNA-binding protein HU |
| -102 | SERP1041 | *hup* | DNA-binding protein HU |
| -22 | SERP1044 | *rpsA* | 30S ribosomal protein S1 |
| -107 | SERP1044 | *rpsA* | 30S ribosomal protein S1 |
| -189 | SERP1044 | *rpsA* | 30S ribosomal protein S1 |
| -90 | SERP1051 | *fer* | ferredoxin |
| -29 | SERP1051 | *fer* | ferredoxin |
| -6 | SERP1102 | *gcvT* | glycine cleavage system aminomethyltransferase |
| -49 | SERP1102 | *gcvT* | glycine cleavage system aminomethyltransferase |
| -62 | SERP1103 | *aroK* | shikimate kinase |
| -271 | SERP1119 | *sodA* | superoxide dismutase |
| -387 | SERP1119 | *sodA* | superoxide dismutase |
| -39 | SERP1132 | *glyS* | glycyl-tRNA synthetase |
| -161 | SERP1153 | *rpsT* | 30S ribosomal protein S20 |
| -174 | SERP1182 | *alaS* | alanyl-tRNA synthetase |
| -48 | SERP1193 | *hisS* | histidyl-tRNA synthetase |
| -45 | SERP1196 | *relA-2* | GTP pyrophosphokinase |
| -71 | SERP1208 | *cgtA* | GTPase ObgE |
| -9 | SERP1233 | *hemD* | uroporphyrinogen-III synthase |
| -13 | SERP1237 | *yihA* | ribosome biogenesis GTP-binding protein YsxC |
| -213 | SERP1246 | *thrS* | threonyl-tRNA synthetase |
| -289 | SERP1246 | *thrS* | threonyl-tRNA synthetase |
| -45 | SERP1254 | *polA* | DNA polymerase I |
| -23 | SERP1256 | *phoP* | alkaline phosphatase synthesis transcriptional |
| -79 | SERP1256 | *phoP* | alkaline phosphatase synthesis transcriptional |
| -14 | SERP1272 | *ald* | alanine dehydrogenase |
| -189 | SERP1293 | *tyrS* | tyrosyl-tRNA synthetase |
| -157 | SERP1295 | *fhs* | formate--tetrahydrofolate ligase |
| -157 | SERP1296 | *ccpA* | catabolite control protein A |
| -37 | SERP1318 | *leuS* | leucyl-tRNA synthetase |
| -79 | SERP1324 | *putA* | proline dehydrogenase |
| -245 | SERP1336 | *tal* | translaldolase |
| -117 | SERP1347 | *tnpA-2* | Tn554, transposase A |
| -7 | SERP1358 | *menE* | O-succinylbenzoic acid--CoA ligase |
| -267 | SERP1358 | *menE* | O-succinylbenzoic acid--CoA ligase |
| -346 | SERP1358 | *menE* | O-succinylbenzoic acid--CoA ligase |
| -35 | SERP1367 | *hemH* | ferrochelatase |
| -49 | SERP1397 | *sspA* | glutamyl endopeptidase precursor SspA |
| -275 | SERP1397 | *sspA* | glutamyl endopeptidase precursor SspA |
| -23 | SERP1400 | *bcp* | bacterioferritin comigratory protein |
| -44 | SERP1401 | *hemL-2* | glutamate-1-semialdehyde aminotransferase |
| -7 | SERP1411 | *recX* | recombination regulator RecX |
| -145 | SERP1426 | *map* | methionine aminopeptidase |
| -193 | SERP1439 | *gatC* | aspartyl/glutamyl-tRNA amidotransferase subunit |
| -171 | SERP1440 | *putP* | proline permease |
| -16 | SERP1446 | *purB* | adenylosuccinate lyase |
| -53 | SERP1446 | *purB* | adenylosuccinate lyase |
| -185 | SERP1456 | *aldA-1* | aldehyde dehydrogenase |
| -99 | SERP1456 | *aldA-1* | aldehyde dehydrogenase |
| -55 | SERP1460 | *blaR1-1* | regulatory protein BlaR1 |
| -38 | SERP1461 | *blaZ-1* | beta-lactamase |
| -26 | SERP1484 | *groEL* | molecular chaperone GroEL |
| -157 | SERP1485 | *groES* | co-chaperonin GroES |
| -322 | SERP1487 | *sdrH* | sdrH protein |
| -5 | SERP1492 | *agrC* | accessory gene regulator protein C |
| -237 | SERP1665 | *ilvD* | dihydroxy-acid dehydratase |
| -35 | SERP1680 | *rsbU* | sigma factor B regulator protein |
| -94 | SERP1680 | *rsbU* | sigma factor B regulator protein |
| -180 | SERP1680 | *rsbU* | sigma factor B regulator protein |
| -186 | SERP1680 | *rsbU* | sigma factor B regulator protein |
| -99 | SERP1690 | *ddl* | D-alanyl-alanine synthetase A |
| -72 | SERP1702 | *sceD* | sceD protein |
| -114 | SERP1702 | *sceD* | sceD protein |
| -212 | SERP1702 | *sceD* | sceD protein |
| -347 | SERP1702 | *sceD* | sceD protein |
| -26 | SERP1710 | *atpG* | ATP synthase F0F1 subunit gamma |
| -68 | SERP1710 | *atpG* | ATP synthase F0F1 subunit gamma |
| -41 | SERP1726 | *tdk* | thymidine kinase |
| -91 | SERP1726 | *tdk* | thymidine kinase |
| -120 | SERP1726 | *tdk* | thymidine kinase |
| -170 | SERP1726 | *tdk* | thymidine kinase |
| -202 | SERP1726 | *tdk* | thymidine kinase |
| -99 | SERP1727 | *rpmE2* | 50S ribosomal protein L31 |
| -195 | SERP1728 | *rho* | transcription termination factor Rho |
| -104 | SERP1731 | *murAB* | UDP-N-acetylglucosamine |
| -211 | SERP1731 | *murAB* | UDP-N-acetylglucosamine |
| -37 | SERP1732 | *fbaA* | fructose-bisphosphate aldolase |
| -153 | SERP1752 | *manA-1* | mannose-6-phosphate isomerase |
| -25 | SERP1760 | *glmS* | glucosamine--fructose-6-phosphate |
| -69 | SERP1795 | *lacA* | galactose-6-phosphate isomerase subunit LacA |
| -94 | SERP1795 | *lacA* | galactose-6-phosphate isomerase subunit LacA |
| -267 | SERP1796 | *lacR* | lactose phosphotransferase system repressor |
| -269 | SERP1796 | *lacR* | lactose phosphotransferase system repressor |
| -91 | SERP1799 | *rplM* | 50S ribosomal protein L13 |
| -93 | SERP1799 | *rplM* | 50S ribosomal protein L13 |
| -109 | SERP1844 | *femX* | femX protein |
| -257 | SERP1844 | *femX* | femX protein |
| -225 | SERP1849 | *sarV* | accessory regulator V |
| -4 | SERP1856 | *moaC* | molybdenum cofactor biosynthesis protein MoaC |
| -181 | SERP1869 | *ureA* | urease subunit gamma |
| -29 | SERP1876 | *sarR* | accessory regulator R |
| -118 | SERP1876 | *sarR* | accessory regulator R |
| -260 | SERP1876 | *sarR* | accessory regulator R |
| -341 | SERP1876 | *sarR* | accessory regulator R |
| -132 | SERP1919 | *hutG* | formimidoylglutamase |
| -143 | SERP1919 | *hutG* | formimidoylglutamase |
| -52 | SERP1924 | *rpiA* | ribose-5-phosphate isomerase A |
| -317 | SERP1926 | *galM* | aldose 1-epimerase |
| -251 | SERP1935 | *gltS* | sodium:glutamate symporter |
| -24 | SERP1948 | *tcaA* | tcaA protein |
| -56 | SERP1948 | *tcaA* | tcaA protein |
| -41 | SERP1960 | *tagF* | teichoic acid biosynthesis protein F |
| -189 | SERP1960 | *tagF* | teichoic acid biosynthesis protein F |
| -48 | SERP1976 | *gltT* | proton/sodium-glutamate symport protein |
| -90 | SERP1987 | *narG* | respiratory nitrate reductase, alpha subunit |
| -145 | SERP1987 | *narG* | respiratory nitrate reductase, alpha subunit |
| -153 | SERP1987 | *narG* | respiratory nitrate reductase, alpha subunit |
| -169 | SERP1990 | *nirB* | nitrite reductase [NAD(P)H], large subunit |
| -19 | SERP2007 | *gpmA* | phosphoglyceromutase |
| -30 | SERP2007 | *gpmA* | phosphoglyceromutase |
| -206 | SERP2007 | *gpmA* | phosphoglyceromutase |
| -144 | SERP2012 | *garK* | glycerate kinase 2 |
| -16 | SERP2012 | *garK* | glycerate kinase 2 |
| -12 | SERP2012 | *garK* | glycerate kinase 2 |
| -223 | SERP2014 | *bcr* | bicyclomycin resistance protein |
| -71 | SERP2026 | *lrgA* | murein hydrolase regulator LrgA |
| -36 | SERP2056 | *galU* | UTP-glucose-1-phosphate uridylyltransferase |
| -162 | SERP2099 | *xylB* | D-xylulose kinase |
| -238 | SERP2107 | *capB* | capB protein |
| -329 | SERP2107 | *capB* | capB protein |
| -396 | SERP2107 | *capB* | capB protein |
| -60 | SERP2144 | *pyrD* | dihydroorotate dehydrogenase 2 |
| -213 | SERP2156 | *ldh* | L-lactate dehydrogenase |
| -4 | SERP2166 | *fdaB* | fructose-1,6-bisphosphate aldolase |
| -37 | SERP2168 | *mqo-2* | malate:quinone oxidoreductase |
| -56 | SERP2168 | *mqo-2* | malate:quinone oxidoreductase |
| -328 | SERP2177 | *betB* | betaine aldehyde dehydrogenase |
| -131 | SERP2242 | *ipdC* | indole-3-pyruvate decarboxylase |
| -81 | SERP2242 | *ipdC* | indole-3-pyruvate decarboxylase |
| -156 | SERP2250 | *arcA* | arginine deiminase |
| -106 | SERP2252 | *sepA* | extracellular elastase precursor |
| -119 | SERP2252 | *sepA* | extracellular elastase precursor |
| -302 | SERP2252 | *sepA* | extracellular elastase precursor |
| -78 | SERP2292 | *icaR* | intercellular adhesion regulator |
| -73 | SERP2293 | *icaA* | N-glycosyltransferase |
| -259 | SERP2297 | *gehC* | lipase |
| -119 | SERP2312 | *mqo-3* | malate:quinone oxidoreductase |
| -97 | SERP2312 | *mqo-3* | malate:quinone oxidoreductase |
| -2 | SERP2331 | *rarD* | rarD protein |
| -169 | SERP2346 | *gldA* | glycerol dehydrogenase |
| -57 | SERP2347 | *bioB* | biotin synthase |
| -145 | SERP2347 | *bioB* | biotin synthase |
| -16 | SERP2366 | *pflB* | formate acetyltransferase |
| -357 | SERP2366 | *pflB* | formate acetyltransferase |
| -44 | SERP2388 | *gehD* | lipase |
| -96 | SERP2388 | *gehD* | lipase |
| -31 | SERP2392 | *bhp* | cell wall associated biofilm protein |
| -88 | SERP2392 | *bhp* | cell wall associated biofilm protein |
| -240 | SERP2396 | *bioD* | dethiobiotin synthase |
| -245 | SERP2396 | *bioD* | dethiobiotin synthase |
| -37 | SERP2398 | *aap* | accumulation associated protein |
| -99 | SERP2398 | *aap* | accumulation associated protein |
| -338 | SERP2398 | *aap* | accumulation associated protein |
| -384 | SERP2398 | *aap* | accumulation associated protein |
| -109 | SERP2463 | *cas1* | CRISPR-associated Cas1 family protein |
| -179 | SERP2463 | *cas1* | CRISPR-associated Cas1 family protein |
| -391 | SERP2463 | *cas1* | CRISPR-associated Cas1 family protein |
| -167 | SERP2474 | *hsdR* | type I restriction-modification system, R |
| -16 | SERP2520 | *mecR1* | methicillin-resistance regulatory protein MecR1 |
| -70 | SERP2521 | *mecA* | penicillin-binding protein 2' |
| -197 | SERP2530 | *yycJ* | metallo-beta-lactamase YycJ |
| -292 | SERP2530 | *yycJ* | metallo-beta-lactamase YycJ |
| -26 | SERP2534 | *yycF* | DNA-binding response regulator YycF |
| -223 | SERP2544 | *hlb* | beta-hemolysin |
| -154 | SERP2544 | *hlb* | beta-hemolysin |
| -262 | SERP2545 | *serS* | seryl-tRNA synthetase |
| -367 | SERP2545 | *serS* | seryl-tRNA synthetase |
| -15 | SERP2552 | *dnaN* | DNA polymerase III subunit beta |
| -347 | SERP2552 | *dnaN* | DNA polymerase III subunit beta |
